# Additive reductions in zebrafish *PRPS1* activity result in a spectrum of deficiencies modeling several human *PRPS1*-associated diseases

**DOI:** 10.1038/srep29946

**Published:** 2016-07-18

**Authors:** Wuhong Pei, Lisha Xu, Gaurav K. Varshney, Blake Carrington, Kevin Bishop, MaryPat Jones, Sunny C. Huang, Jennifer Idol, Pamela R. Pretorius, Alisha Beirl, Lisa A. Schimmenti, Katie S. Kindt, Raman Sood, Shawn M. Burgess

**Affiliations:** 1 Translational and Functional Genomics Branch, National Human Genome Research Institute, Bethesda, MD 20892, USA; 2 Cancer Genetics and Comparative Genomics Branch, National Human Genome Research Institute, Bethesda, MD 20892, USA; 3 Department of Biology, Hanover College, Hanover, IN, 47243, USA; 4 Section on Sensory Cell Development and Function, National Institute on Deafness and Other Communication Disorders, Bethesda, MD 20892, USA; 5 Departments of Otorhinolaryngology and Clinical Genomics, Mayo Clinic, Rochester, MN 55905, USA

## Abstract

Phosphoribosyl pyrophosphate synthetase-1 (*PRPS1*) is a key enzyme in nucleotide biosynthesis, and mutations in *PRPS1* are found in several human diseases including nonsyndromic sensorineural deafness, Charcot-Marie-Tooth disease-5, and Arts Syndrome. We utilized zebrafish as a model to confirm that mutations in *PRPS1* result in phenotypic deficiencies in zebrafish similar to those in the associated human diseases. We found two paralogs in zebrafish, *prps1a* and *prps1b* and characterized each paralogous mutant individually as well as the double mutant fish. Zebrafish *prps1a* mutants and *prps1a;prps1b* double mutants showed similar morphological phenotypes with increasingly severe phenotypes as the number of mutant alleles increased. Phenotypes included smaller eyes and reduced hair cell numbers, consistent with the optic atrophy and hearing impairment observed in human patients. The double mutant also showed abnormal development of primary motor neurons, hair cell innervation, and reduced leukocytes, consistent with the neuropathy and recurrent infection of the human patients possessing the most severe reductions of PRPS1 activity. Further analyses indicated the phenotypes were associated with a prolonged cell cycle likely resulting from reduced nucleotide synthesis and energy production in the mutant embryos. We further demonstrated the phenotypes were caused by delays in the tissues most highly expressing the *prps1* genes.

Phosphoribosyl pyrophosphate synthetase-1 (PRPS1) is an enzyme functioning at the earliest steps of nucleotide biosynthesis, catalyzing the phosphoribosylation of ribose-5-phosphate to phosphoribosyl pyrophosphate (PRPP). PRPP is an essential component for both the *de novo* and salvage pathway synthesis of purine and pyrimidine nucleotides. Biosynthesis of these nucleotides is precisely regulated within individual cells. The resulting nucleotides play critical roles in various biological processes, including serving as the building blocks of DNA and RNA, participating in cell signaling, acting as co-factors for enzymatic reactions, and providing energy for metabolism^1^,^2^.

In humans, the *PRPS1* gene is located on the X chromosome. Hemizygous mutations in *PRPS1* are associated with a variety of X-linked diseases that primarily affect males^3^,^4^,^5^. In recent years, there have been a growing number of reported human patients carrying *PRPS1* mutations^4^,^5^,^6^,^7^,^8^. To date, approximately 25 pathogenic *PRPS1* mutations have been reported, with 8 of these mutations discovered since 2014. All of the reported *PRPS1* human mutations are missense alleles in the coding region of the *PRPS1* gene and the majority of them cause a reduction in PRPS1 activity^4^,^7^. Although there is considerable variation in the genotype-phenotype correlation, in general the phenotypic severity is related to the degree of reduction in PRPS1 activity. Modest reductions in PRPS1 activity are associated with X-linked non-syndromic sensorineural deafness where patients have post-lingual progressive hearing loss (Deafness, X-linked 1 (DFNX1), MIM 304500). A moderate reduction of PRPS1 activity is associated with X-linked Charcot-Marie-Tooth where patients have hearing impairment, together with optic atrophy and peripheral neuropathy (CMTX5, MIM 311070). More severe reductions of PRPS1 activity are associated with Arts Syndrome where patients have not only hearing impairment, optic atrophy, and peripheral neuropathy, but also central neuropathy and a deficient immune response (MIM 301835). The most severe form of PRPS1 deficiency is associated with a disorder whose patients have central neuropathy such as severe intellectual disability and spastic quadraparesis, along with prenatal growth retardation and dysmorphic facial features^6^. PRPS1 hyperactivity can also result in pathology. Mutations that increase PRPS activity have been linked to gout^9^ and to chemotherapy resistance in cancer^10^. Many of the *PRPS1*-related patients presented clinical manifestations at an early age^6^,^11^,^12^, suggesting *PRPS1*-related phenotypes are associated with defects in development. It remains unclear how PRPS1 deficiency contributes to diseased tissue functionality, and why the phenotypes arise in specific tissues.

No animal model had been made to confirm *PRPS1* mutations caused the disease phenotypes seen in humans, although several animal models exist for mutations in the genes downstream of PRPS1 in the nucleotide synthesis pathway. For example, mouse mutations in *Ampd* caused defects in brain neurogenesis and a severely shortened lifespan^13^. Zebrafish mutations in *gart* and *paics* affected ocular and pigmentation development^14^. Drosophila mutations in *impdh* and *gmp* lead to defects in axon pathfinding^15^. These studies demonstrated the importance of nucleotide synthesis for a variety of developmental functions, but also emphasized each component of the nucleotide synthesis pathway appears to have phenotypes relating to development or pathogenesis.

Here we used zebrafish to model the *PRPS1*-associated genetic diseases. Zebrafish has two paralogs of *prps1*, *prps1a* and *prps1b*. We examined their gene expression in the early embryo and created zebrafish mutants carrying mutations for each individually or both together. We also demonstrated a link between the degree of gene inactivation and phenotype severity, which is consistent with observations made in human patients where the level of PRPS1 activity was correlated with disease severity.

## Results

### Zebrafish *prps1a* and *prps1b* are **relatively enriched** in the embryonic **brain**, inner ear, and caudal hematopoietic tissue

**Figure 1:**
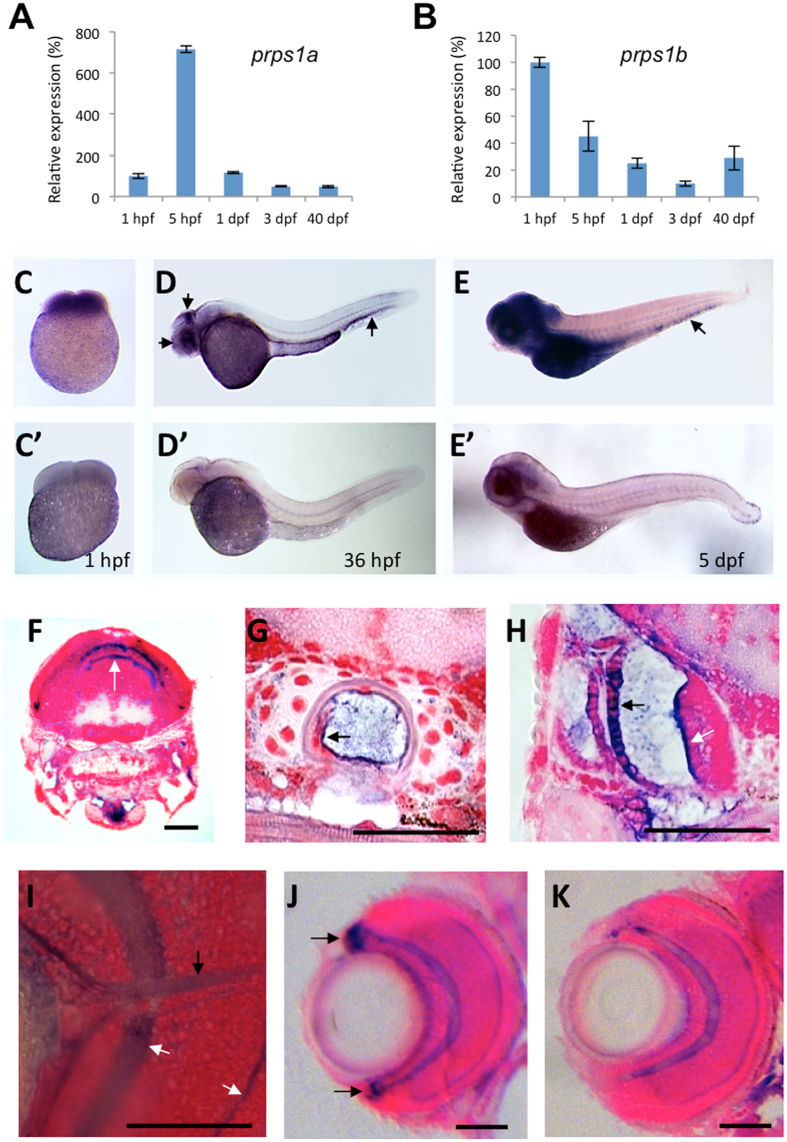
*prps1a* and *prps1b* expression in early zebrafish development. (**A,B**) qRT-PCR analysis of *prps1a* (**A**) and *prps1b* (**B**) expression at five developmental stages. 20 embryos at stages 1 hpf, 5 hpf, 1 dpf, 3 dpf, or 3 juvenile fish at 40 dpf were pooled together for total RNA extraction, and then qRT-PCR analysis. The internal reference used for qPCR is β–actin, whose expression may vary from maternal to zygotic stages but suffices to compare temporal expression differences between genes. Graphs show the mean and standard deviation, with the relative expression level at each timepoint normalized to 1 hpf (100%). (**C–E**) Whole mount *in situ* hybridization analysis of *prps1a* expression at the 1 hpf (**C**), 36 hpf (**D**), and 5 dpf (**E**). 15 embryos per group were used for *in situ* hybridization analysis. Representative images are shown for the antisense (**C**–**E**) and sense probes (C’–E’). Three arrows in D point to the enrichment in the eye, tectum and hematopoetic tissue. Images in (**E**,E’) are from an extended staining to show the expression in the hematopoietic tissue (arrow in **E**). (**F–I**) Histological sections reveal *prps1a* expression in the brain (**F**), notochord (**G**), inner ear (**H**), and optic nerve (**I**). *prps1a* and *prps1b* expression was very similar, therefore representative *prps1a* images are shown. White arrow in F points to the enrichment in two stripes in the dorsal brain. Black arrow in G points to the enrichment in cells of the notochord. Black arrow and white arrow in H point to in the outer otic vesicle epithelium and the sensory epithelium of cristae of the inner ear, respectively. White arrows in I point to the retinal inner and outer plexiform layers, and the black arrow in I points to the optic nerve, all of which show relatively elevated levels of *prps1a* expression. (**J,K**) Histological sections reveal enriched *prps1a* expression in the retinal cilliary marginal zone, black arrows in J points to the expression of *prps1a* in ciliary marginal zone (**J**), but *prps1b* is not expressed in the CMZ (**K**). Images in (**F–K**) were obtained from *in situ* stained 5-day old embryos that were transversely sectioned and then counterstained with nuclear fast red. Scale bars in (**F–K**), 50 μm.

Humans and mice have a single copy of the *PRPS1* gene, while zebrafish has two paralogs, *prps1a* (chromosome 5) and *prps1b* (chromosome 14). To study how these two paralogs are transcriptionally regulated during zebrafish embryonic development, we compared their expression levels at different developmental stages using quantitative reverse transcription polymerase chain reaction (qRT-PCR) and found the two paralogs had distinct expression profiles (Fig. 1A,B). For *prps1a*, a low level expression was detected at 1 hour post fertilization (hpf), prior to the onset of zygotic transcription, indicating that the mRNA was maternally expressed. A dramatic increase of expression was detected from 5 hpf. The expression decreased and was maintained at a lower level until at 40 dpf as the fish entered a juvenile age. In contrast, *prps1b* displayed a more modest change in the level of expression with the highest expression detected at 1 hpf and a lower level present and maintained at other ages. Like *prps1a*, *prps1b* expression was relatively low at 40 dpf. These data suggest *prps1a* and *prps1b* are required at higher levels for the early ages of embryo development and drop to a more maintenance level as the zebrafish matures.

We then performed whole-mount *in situ* hybridization (WISH) to identify spatial expression patterns. Probes were designed to detect each of the two paralogs separately. We found the transcripts of both paralogs had very similar tissue distribution. Both *prps1a* and *prps1b* were ubiquitously expressed in 4-cell stage embryos. At 36 hpf, both transcripts were more highly expressed in the retina, brain tectum and yolk syncytial layer. The brain and retinal enrichment of expression persisted through 5 days of development, when increased expression was also visible in other internal organs and caudal hematopoietic tissue (Fig. 1C–E, Suppl. Fig. 1C–E).

Histological sectioning of *in situ* stained 5-day-old embryos revealed that the tissue location of *prps1a* and *prps1b* were largely similar but with some important differences. Both were enriched in the dorsal regions of the brain, notochord, as well as the cristae and semi-circular canals of the inner ear (Fig. 1F–H, Suppl. Fig. 1A,B). Although both were enriched in the retinal plexiform layers and optic nerve (Fig. 1I), it was *prps1a* (Fig. 1J), not *prps1b* (Fig. 1K), that was enriched in the ciliary marginal zone (CMZ), a retinal stem cell zone with highly proliferating cells. The expression difference in the CMZ suggests a potential functional divergence between these two paralogs in retinal growth and/or development.

### Generation of *prps1a* and *prps1b* zebrafish mutants

**Figure 2:**
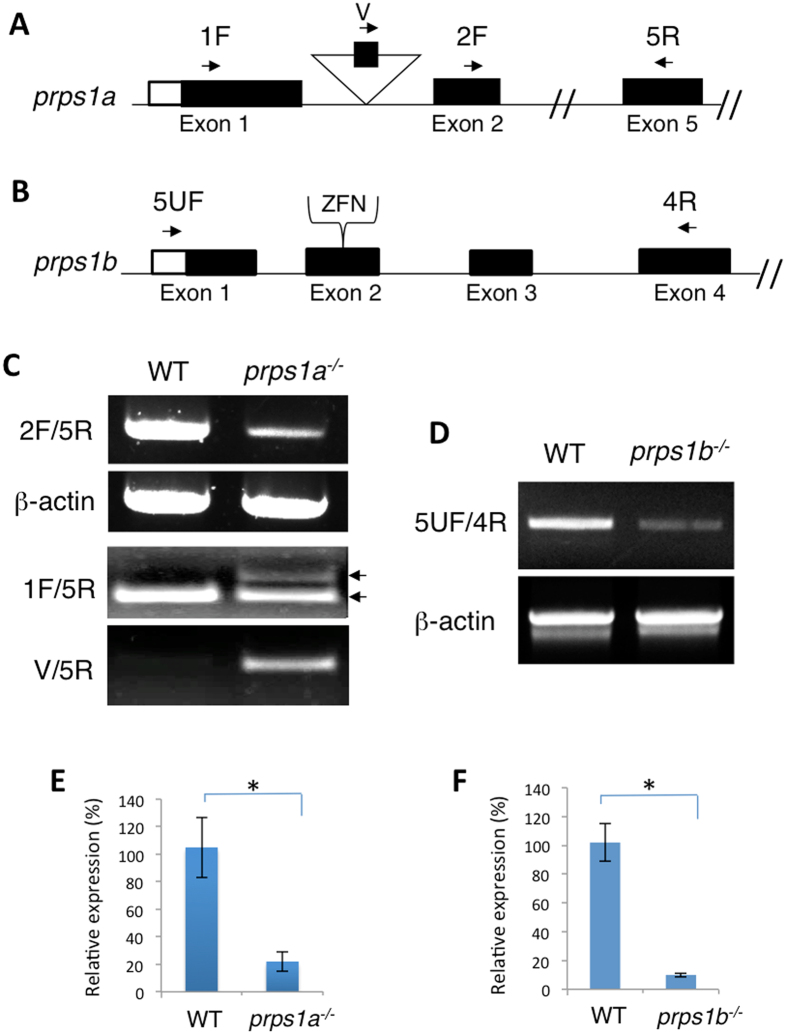
Generation of *prps1a* and *prps1b* genetic mutations. (**A**,**B**) Schematic diagram of *prps1a* and *prps1b* mutations. The *prps1a*^la015591^ mutant has a retroviral integration in the first intron of the gene that destabilizes the mRNA. The *prps1b* mutant has a small indel created by zinc finger nuclease targeted mutagenesis. The line represents genomic DNA. Open boxes represent the 5′UTR region. Solid boxes represent coding exons. The double slashes in the genomic DNA represent a break in the DNA continuity. Arrows mark the locations and orientations of the primers used. The triangle in A indicates the viral integration site. The target of zinc finger nuclease is indicated in (**B**). (**C,D**) RT-PCR analysis of mRNA expression in WT and homozygous mutants. The cDNA was extracted from 10 embryos per group at 3 dpf. Beta-actin is used as an internal reference. Amplicons from 1F/5R and V/5R primer pairs were obtained by extended amplification in order to visualize the *prps1a* mutant mRNA that contains the retroviral exon. Two black arrows in C point to the exon-trapped mutant (top arrow) and wild-type (bottom arrow) of *prps1a* mRNA present in *prps1a* mutant. (**E,F**) Quantification of the reduction of *prps1a* (**E**) and *prps1b* (**F**) transcripts in the homozygotic embryos by qRT-PCR. Approximately 20% of the normal RNA level is present in *prps1a*^*−/−*^. As *prps1b* is a frame-shift mutation, the reduction in mRNA level is likely due to nonsense mediated decay and the allele is presumed to be a null or near null. The double mutants have less than 20% of the normal total level of functional *prps1* mRNA.

To study the function of *prps1a* and *prps1b* in zebrafish, we generated mutations in each paralog. The *prps1a* mutation (*prps1a*^*la015591*^) was generated by retroviral integration^16^, containing a 6 kb retroviral DNA insertion in the first intron of the *prps1a* gene (Fig. 2A). RT-PCR analysis showed that there was a low residual level of *prps1a* expression in the homozygous mutant embryos (Fig. 2E). Because the retroviral DNA contained a gene trap^17^, the internal exon of the gene trap could be spliced into the *prps1a*^*la015591*^ mRNA. To test this, we used a primer (V primer) flanking the internal exon and found the residual low level of *prps1a* expression in *prps1a*^*la015591*^ homozygotes was comprised of both wild-type *prps1a* mRNA and mutant *prps1a* mRNA containing the internal exon derived from retroviral DNA (Fig. 2C,E). The *prps1b*^*hg19*^ mutation was generated by zinc finger nuclease (ZFN, designed by Sigma Aldrich), targeting the second exon of the gene (Fig. 2B). Three frame-shift mutations were analyzed (*prps1b*^*hg19*^*, prps1b*^*hg20*^*, prps1b*^*hg21*^; Suppl. Fig. 1F). All of them showed a comparable level of reduced mRNA expression in the mutant embryos, with the data for a deletion of 5 base pair shown in Fig. 2D,F. As these mutations are all frame-shift deletions, the reduction in mRNA expression is likely the result of nonsense-mediated decay of the transcript.

### *prps1a* mutants displayed defects in the eye, the neuromast hair cells, and in pigmentation

**Figure 3:**
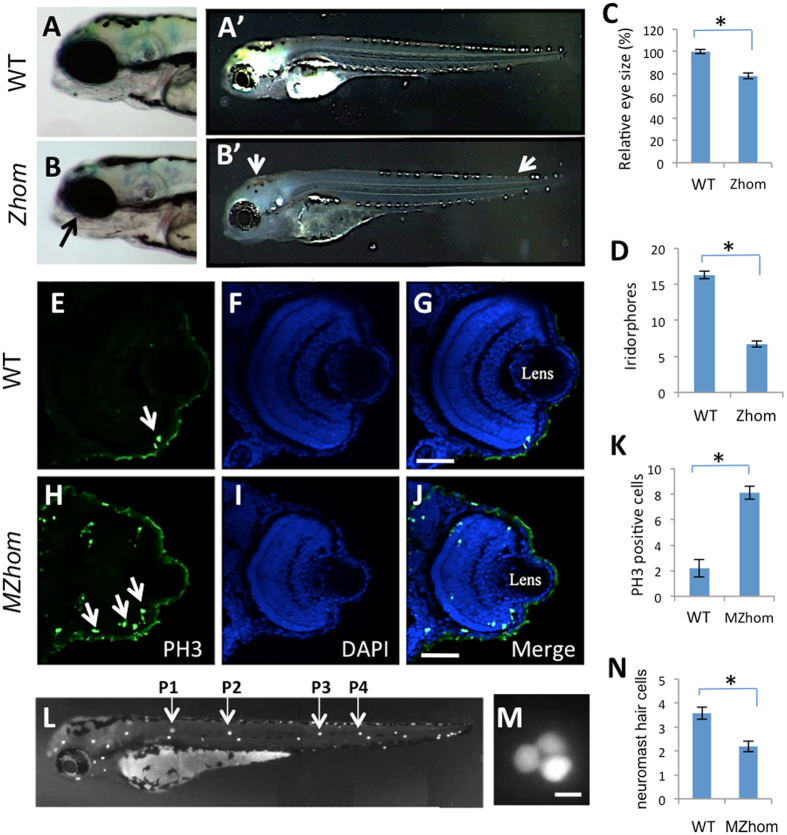
The *prps1a* mutation causes a reduction in the eye, a loss of iridophores, and a reduction in neuromast hair cells. (**A,B**) Eye phenotype in the control (**A**) and *prps1a* Zhom (**B**) embryos at 3 dpf. Black arrow in B points to the slightly smaller eye in the Zhom mutant. (A’,B’) Iridophore phenotype in control (A’) and *prps1a* Zhom (B’) embryos at 3 dpf. White arrows in B’ point to the areas where iridophores are reduced in different tissues. (**C,D**) Quantification of the reduction in eye size (**C**) and iridophores (**D**). Eye area was calculated by image J. The numbers of iridophores were obtained by counting the iridophores in the dorsal and ventral side of the trunk area above the yolk extension. The reduction was significant in the eye size (n = 8, p < 0.001) and iridophores (n = 12, p < 0.001). (**E**–**J**) Immunohistochemical staining of phosphorylated histone H3 Ser10 in the control and MZhom embryos at 3 dpf. Columns from left to right show the representative images from the staining of phosphorylated histone H3 Ser10, DAPI, and the merged image. White arrows in H point to representative positive cells. (**K**) Quantification of phosphorylated histone H3 cells in the retina. The increased number of H3 Ser10 positive cells in the MZhom embryos is significant (n = 5 for Ctrl, n = 7 for MZhom, p < 0.001). (**L**–**M**) Fluorescent images of a wild-type Yopro-1 stained embryo at 3 dpf showing the location of neuromasts (**L**) and a higher magnification of the P1 neuromast (**M**). Hair cells in P1–P4 neuromasts were counted for *prps1a* mutants and control siblings. (**N**) *prps1a* homozygous mutants have a reduced number of neuromast hair cells at 3 dpf. The reduction is significant (n = 10, p < 0.001). Scale bars: 50 μm in (**G,J**); 10 μm in (**M**).

For the *prps1a* mutation, we studied the morphological phenotypes of zygotic homozygotes (Zhom), as well as maternal-zygotic homozygotes (MZhom). Since wild-type (WT) and heterozygous carriers showed indistinguishable morphology (data not shown), they were grouped together as the control embryos. *prps1a* Zhom showed smaller eyes and reduced iridophore pigmentation. Both phenotypes were identifiable starting from 3 dpf (Fig. 3A–D), and persisted throughout embryonic development. A combination of small-eye and reduced iridophore phenotypes was found in 25/25 of the Zhom embryos and 0/25 of the control embryos, making it an accurate identifier for homozygous mutants. The MZhom mutants displayed more severe eye and iridophore phenotypes. The small-eye phenotype was detectable starting at 1 dpf and persisted through early development (Suppl. Fig. 2A). The MZhom also displayed a further reduction in iridophores (Suppl. Fig. 2B). Interestingly, both the reduced eye and iridophore phenotypes disappeared when the MZhom reached adulthood (data not shown), suggesting a recovery of phenotypes later in development.

To test the severity of the phenotype for the *prps1a* viral mutant, we knocked down *prps1a* by CRISPR/Cas9, targeting exon 1 of the gene. Injection of Cas9 mRNA alone caused no somatic mutations and no morphological phenotypes, however, co-injection of Cas9 mRNA and a *prps1a* single guide RNA with high targeting activity caused essentially 100% somatic mutation at the target site. In all embryos analyzed, the phenotypes were similar but often slightly more severe than those of the *prps1a* viral insertional mutant, including small eyes and reduced iridophores (Suppl. Fig. 3, Suppl. Table 1).

Histological sectioning was performed on the Zhom and MZhom at 3 dpf and 6 dpf. The Zhom displayed a delay in retinal development and lamination at 3 dpf, however the delay was partially recovered by 6 dpf (Suppl. Fig. 2C). Sectioning analysis also confirmed that the MZhom had more severe phenotypes than the Zhom. At 3 dpf, the MZhom showed no visible retinal lamination together with a thinning of the retinal pigment epithelium layer. From 3 dpf to 6 dpf, the MZhom were able to establish retinal lamination. At 6 dpf, the MZhom had a normal-looking retinal pigment epithelium layer, and their retinal lamination was comparable to that of the Zhom of the same age (Suppl. Fig. 2D).

To identify the reason for the retinal phenotype in the MZhom fish, immunohistochemistry was performed on 3 dpf embryos staining the phosphorylated histone H3 Ser10, which specifically marks mitotic cells. In the control embryos, a small number of retinal cells were dividing and the dividing cells were located along the peripheral retina (Fig. 3E–G). In the MZhom, an increased number of retinal cells were in the process of dividing and these dividing cells spread more toward the central retina (Fig. 3H–K) despite the fact that there were fewer total cells in the retina compared to the wild-type. These data suggest the *prps1a* mutation leads to a prolonged cell cycle. Although we did not perform further tests, the increase in the number of cells observed in mitosis may be the result of a shift in the length of S-phase progression as was noted in zebrafish mutations for *gart* and *paics*, two genes downstream of *prps1* in nucleotide synthesis^14^.

Hearing impairment and ataxia are characteristics of *PRPS1*-associated diseases in humans. Hair cells of the inner ear are responsible for both hearing and balance. In addition to the hair cells of the inner ear, zebrafish also possesses a second organ that contains hair cells known as the lateral line neuromast (Fig. 3L,M). Hair cells in the lateral line are structurally and molecularly similar to the inner ear hair cells. However, neuromast hair cells are at the skin’s surface making them easily accessible for staining and imaging *in vivo*. Yopro-I staining of neuromast hair cells revealed that the *prps1a* MZhom embryos had a significantly reduced number of hair cells at 3 dpf (Fig. 3N). However, the reduction was no longer detectable at 5 dpf (Suppl. Fig. 2E). Since neuromast hair cells typically have a peak of growth between 3–5 dpf, these data suggest the reduced number of neuromast hair cells in the MZhom is due to a delay in hair cell differentiation, which can eventually “catch up” to normal developmental levels in the larvae when growth normally slows.

### *prps1b* mutants showed no overt phenotype

Both zygotic and maternal-zygotic homozygotes were examined for the *prps1b* mutations. None of the three frame-shifted mutations caused overt embryonic phenotypes, or an alteration in the development of neuromast hair cells (data not shown). *prps1b* mutants survived to adulthood, had a normal life span, and were able to normally reproduce. Since the 5 base pair deletion (*prps1b*^*hg19*^) resulted in a premature stop codon and a second stop codon 21 nucleotides downstream (Suppl. Fig. 1F,G), it was chosen for the generation of *prps1a;prps1b* double mutants.

### *prps1a;prps1b* double mutants displayed more severe defects in the eye, pigmentation, and the neuromast hair cells

**Figure 4:**
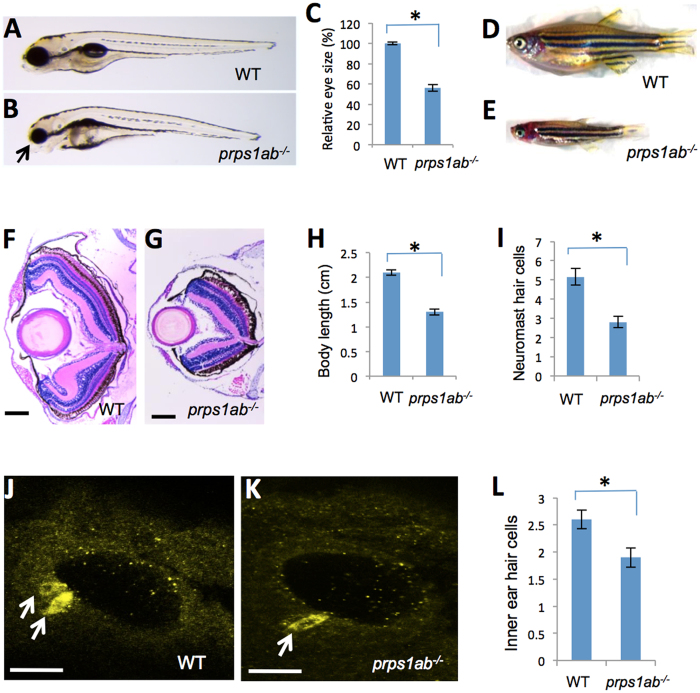
*prps1a;prps1b* double mutant displays defects in the eye, craniofacial structure, general growth, neuromast and inner ear hair cells. (**A,B**) Eye phenotype of the double mutant. Images show genetically-related wild-type (**A**) and the double mutant (**B**) at 5 dpf. Arrow in B points to the small eye and abnormal craniofacial structure of the double mutant. Most double mutants fail to inflate the swim bladder. (**C**) Quantification of the reduction in the eye size. Eye area is calculated using Image J. The reduction is significant (n = 9, p < 0.001). (**D,E**) Morphology of wild-type (**D**) and the double mutant fish (**E**) at 35 dpf, only 5% of the double mutant fish survive to this age. (**F,G**) Retinal lamination of wild-type (**F**) and the double mutant (**G**) at 35 dpf. The double mutant had a poorly formed retinal inner plexiform layer and has an under-developed retinal pigment epithelial layer. (**H**) Quantification of the body length at 35 dpf. The reduction in the double mutant is significant (n = 6, p < 0.001). (**I**) Quantification of the neuromast hair cells in the double mutants at 3 dpf. The reduction in the double mutant is significant (n = 9 for wild-type, n = 11 for the double mutant, p < 0.001). (**J**,**K**) Inner ear hair cells in the embryos at 32 hpf were stained with a mixture of myosin-VIIa and hair cell soma-1 antibodies. 14 embryos per group were used for inner ear hair cell analysis. Representative images are shown. White arrows indicate positive staining of hair cells. (**L**) Quantification of the reduction of inner ear hair cells. The reduction in the double mutants is significant (n = 14, p = 0.003). For all graphs, the average and s.e.m are shown. *prps1ab*^*−/−*^ indicates the *prps1a:prps1b* double mutants. Scale bars: 200 μm in (**F,G**); 50 μm in (**J,K**).

It was hypothesized that since zebrafish have two *prps1* paralogs, a more severe *prps1* loss-of-function phenotype could be assessed in double mutant fish. To examine the *prps1a*;*prps1b* double mutant phenotypes, we analyzed the embryos generated from an in-cross of a pair of adult zebrafish carrying a heterozygous mutation in *prps1a* and homozygous mutation for *prps1b*. The resulting offspring were 25% *prps1a;prps1b* double mutants and showed phenotypes more severe than that of either the *prps1a* mutants or *prps1b* mutants alone. The double mutants at 5 dpf had dysmorphic craniofacial features and very small eyes (Fig. 4A–C). Beyond the reduction in iridophores seen in the *prps1a* homozygous mutants (Suppl. Fig. 2B), the double mutants also displayed a reduction of melanocytes in both the head and the eyes late the first day after fertilization (Suppl. Fig. 4A) although the reduction soon recovered to wild-type levels by 2 dpf (data not shown). In contrast to the healthy and fertile adult *prps1a* or *prps1b* homozygotes, the double mutant showed a dramatic reduction in body size (Fig. 4D,E,H) with the majority of fish having a life span shorter than 40 days. Despite the early morphological phenotypes, approximately 5% (6 out of 112) of the double mutants survived to adulthood. While the fish survived, there was a clear failure to thrive and all suffered from severely stunted growth and would not breed (Fig. 4E).

Histological sectioning revealed that the retina of the double mutant at 35 dpf was not only smaller, but the lamination was somewhat disorganized with poor neuronal differentiation (Fig. 4F,G). Several features observed in the double mutant retinas included small size, disorganized outer plexiform layer, poorly specified retinal pigment epithelial layer, and reduced thickness of the optic nerve.

Neuromast hair cells were also analyzed in the double mutants. Similar to the *prps1a* MZhom, the double mutants had a significant reduction in the number of neuromast hair cells at 3 dpf (Fig. 4I), but the reduction was not detectable at 5 dpf (data not shown). Since the *prps1b* mutation had no effect on neuromast hair cell development, the similar level of reduction in neuromast hair cells of the *prps1a* mutant and *prps1a;prps1b* double mutants suggests the reduction of neuromast hair cells in the double mutant resulted primarily from the loss of *prps1a* expression.

### Reduced number of inner ear hair cells in *prps1a;prps1b* double mutants

Immunohistochemistry was performed to examine the development of inner ear hair cells in the *prps1a;prps1b* double mutants. Inner ear hair cells were labeled by a combination of two monoclonal antibodies against myosin-VIIa and hair cell soma-1. A significant reduction of inner ear hair cells was observed in the double mutant at 32 hpf and also at 3 dpf (Fig. 4J–L, Supplemental Fig. 4C–E). These data are consistent with the reduction of neuromast hair cells, suggesting *prps1* is required for the normal addition of both neuromast hair cells and inner ear hair cells.

### Abnormal motor neuron development in *prps1a;prps1b* double mutants

**Figure 5:**
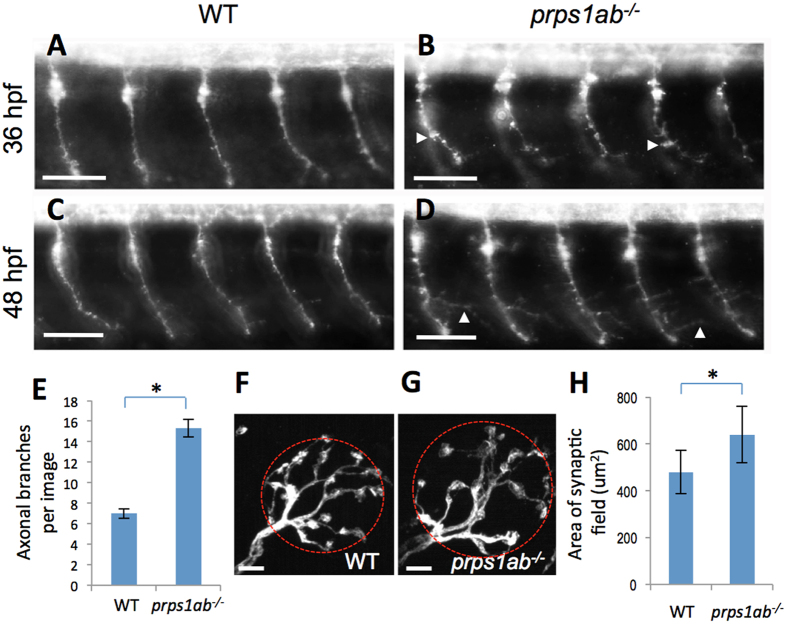
*prps1a:prps1b* double mutants display abnormal morphology in primary motor neuron and neuromast hair cell innervation. (**A**–**D**) Primary motor neurons stained using the znp-1 antibody. (**A,B**) Morphology of motor neuron axons in wild-type (**A**) and double mutant (**B**) embryos at 36 hpf. White arrowheads point to aberrant pseudopodia. The axons in the double mutants were shorter with increased branching compared to wild-type siblings. (**C,D**) Morphology of motor neuron axons in wild-type (**C**) and double mutant (**D**) embryos at 48 hpf. White arrowheads point to elongated branches. 10 embryos are used for each group of staining, with one representative image shown. (**E**) Quantification of axon branching. Y-axis indicates the number of elongated branches per image that frames 5 neurons. The increase in axon branching is significant in the double mutant (n = 10, p < 0.001). (**F–H**) Afferent neurons innervating neuromast hair cells stained with a calretinin antibody. (**F,G**) Morphology of innervating afferent neurons and fibers in wild-type (**F**) and double mutant (**G**) at 6 dpf, red circles are examples of the area calculated. (**H**) Quantification of the innervating areas. The analysis was done with 12 neuromasts from 9 wild-type embryos and 13 neuromasts from 6 double mutants. The area each neuron innervates is significantly greater in the double mutants. The difference between wild-type and double mutant is significant (Tukey’s multiple comparisons, p < 0.01). Scale bars: 50 μm in (**A–D**); 5 μm in (**F,G**).

Neuropathy is a key feature of the human diseases associated with severe reduction of PRPS1 activity. Several neurological symptoms, such as delayed motor development and spastic quadraparesis, suggest a dysfunction in motor neurons that coordinate the movements of the human body and limbs. Znp-1 antibody staining showed primary motor neurons in the *prps1a;prps1b* double mutants had an aberrant, increased branching of the axons. When compared to the control embryos, the double mutant displayed increased pseudopodia at 36 hpf (Fig. 5A,B, white arrowheads), and increased axon branching at 48 hpf (Fig. 5C,D, white arrowheads). While the mechanism for this aberrant branching is unknown, these data indicate depletion of Prps1 in zebrafish impairs axon patterning in the primary motor neurons.

### Abnormal hair cell innervation in *prps1a;prps1b* double mutants

Since hearing impairment is a common symptom shared by several PRPS1-associated diseases, we examined neuromast hair cell innervation as a potential source of pathology. Immunohistochemical staining with antibodies recognizing calretinin revealed there was an expanded area of afferent innervation neurons and fibers for neuromast hair cells in the double mutants (Fig. 5F–H, red circles). The double mutants also showed an increased number of presynaptic ribbon synapses (Suppl. Fig. 5). Further analysis using embryos from earlier stages showed that the increases in presynaptic ribbon synapses were also observed in the younger, wild-type and mutant embryos, indicating these phenotypes are likely due to a developmental delay in the mutants at the later stages. These data suggest loss of Prps1 has some impact on the development of sensory neurons in the lateral line.

### Reduced leukocytes in *prps1a;prps1b* double mutants

**Figure 6:**
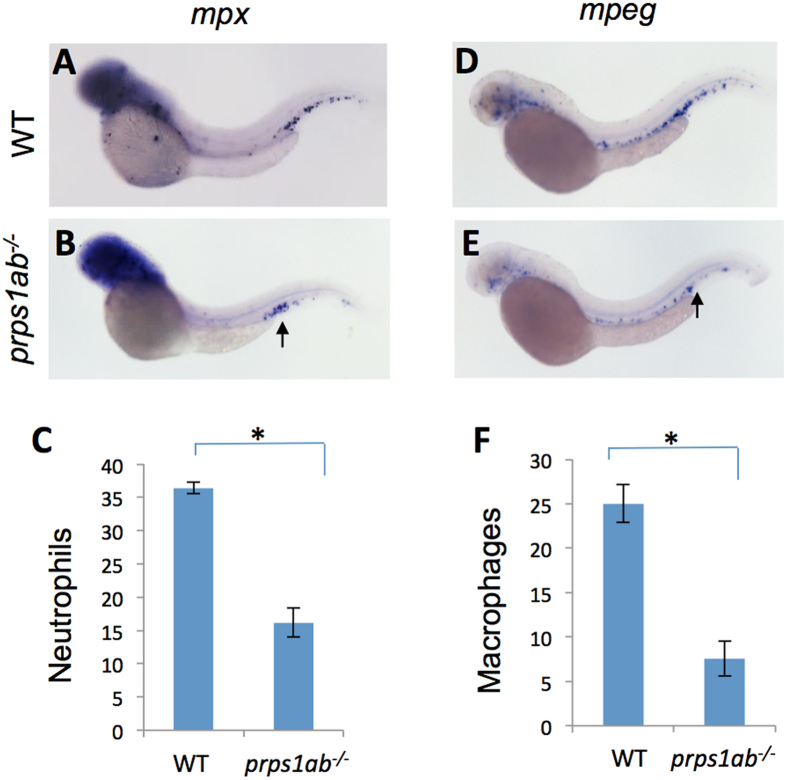
*prps1a:prps1b* double mutants had a reduced number of leukocytes. (**A**–**C**) Double mutant fish had a reduced number of neutrophils. The genetically-related wild-type (**A**) and double mutant (**B**) embryos at 2 dpf were probed for *mpx* (neurtrophil) mRNA expression. Representative lateral view images of embryos are shown. Qualification of the data is shown in (**C**). *prps1ab*^*−/−*^ indicates *prps1a:prps1b* double mutant. (**D**–**F**) Double mutants have a reduced number of macrophages. Wild-type (**D**) and double mutant (**E**) embryos at 2 dpf are probed for *mpeg* mRNA expression (macrophages). Representative images are shown. Quantification data are shown in (**F**). For the quantification, leukocytes in the caudal hematopoietic tissue are counted. Graphs show the average numbers of neutrophils or macrophages per embryo. Error bars show the s.e.m. The double mutants show a significant reduction of both neutrophils and macrophage (n = 10, p < 0.001 for both).

Human patients with Arts Syndrome, the disease associated with severe mutations in the *PRPS1* gene, experience recurrent infections. Based on WISH data, both *prps1a* (Fig. 1E) and *prps1b* (Suppl. Fig. 1E) were enriched in expression in the caudal hematopoietic tissue where leukocytes reside compared to the surrounding tissues. To investigate how the *prps1* mutation is associated with recurrent infection, we studied the development of leukocytes in the double mutant fish. Whole mount *in situ* hybridization analyses were performed using an *mpx* probe that primarily labels neutrophils and an *mpeg1* probe that specifically labels macrophages. The double mutants showed a significant reduction of neutrophils (Fig. 6A–C) and macrophages (Fig. 6D–F), suggesting a requirement of Prps1 activity in normal leukocyte development or maintenance.

Since a small percentage of the double mutants survived to adulthood, we revisited leukocyte development in these adults. Three control adults and three double mutant adults were used for hematopoietic blood cell lineage analysis. No significant difference was found between the control and double mutants for either the myeloid cell lineage that include monocyte/macrophages and neutrophils, or the progenitors that could become myeloid cells (Suppl. Fig. 4F,G). These data suggest these surviving double mutant females recovered from early defects in leukocyte development.

### Partial phenocopy of *prps1a;prps1b* double mutant phenotypes by inhibiting nucleotide biosynthesis

To understand whether the observed phenotypes could be explained by limited nucleotide production due to reduced Prps1 activity in the mutant embryos, we inhibited the synthesis of pyrimidine and purine by pharmacological compounds leflunomide or mycophenolate mofetil, respectively^18^,^19^,^20^. We found leflunomide (pyrimidine inhibition) treatment led to a spectrum of phenotypes resembling those in *prps1* mutants. These phenotypes were a dose-dependent reduction in eye size and iridophores (Suppl. Fig. 6A–C), as previously reported^19^. In addition, leflunomide also caused a dose-dependent reduction in neuromast hair cells (Suppl. Fig. 6D). Increased concentration of leflunomide resulted in additional body axis phenotypes not seen in the *prps1* mutants.

Blocking purine synthesis by mycophenolate mofetil also caused a reduction in eye size, melanocytes, and neuromast hair cells (Suppl. Fig. 7), as seen in leflunomide treatment. However, mycophenolate mofetil also caused additional phenotypes such as heart edema and tail curvature, suggesting pyrimidine synthesis and purine synthesis may have both common and distinct roles in the development of different tissues and in the *prps1* mutants the deficiencies are related to reductions in the synthesis of both pyrimidine and purine. Perhaps the purine biosynthesis pathways have partial compensation feedback in *prps1a;prps1b* mutant fish that are not available when the purine pathway is completely blocked.

### Partial phenocopy of *prps1a;prps1b* double mutant phenotypes by inhibiting ATP production

**Figure 7:**
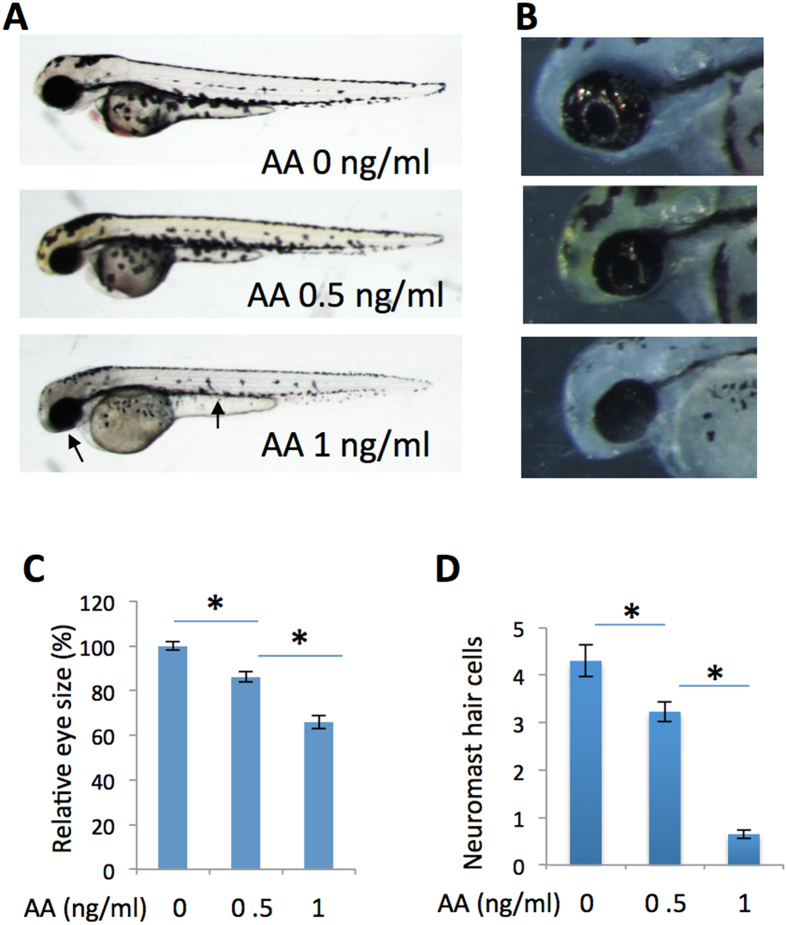
Inhibition of energy ATP production by antimycin A partially phenocopies the *prps1a;prps1b* mutant phenotypes. (**A**) Antimycin A treatment of wild-type embryos produces a dose-dependent reduction in melanocytes and eye size. Antimycin A concentrations are indicated. Arrows point to small eye and melanocyte reduction in the embryo treated with 1 ng/ml of antimycin A. (**B**) Dose-dependent reduction of retinal iridophores in the treated embryos. (**C**) Quantification of eye size reduction. Eye size is measured by Image J. A significant reduction is detected between antimycin A at 0 ng/ml and 0.5 ng/ml (t-test, p < 0.001), and between antimycin A at 0.5 ng/ml and 1 ng/ml (t-test, p = 0.019). (**D**) Dose-dependent reduction of neuromast hair cells in antimycin A-treated embryos. A significant reduction is detected between antimycin A at 0 ng/ml and 0.5 ng/ml (t-test, p = 0.008), and between antimycin A at 0.5 ng/ml and 1 ng/ml (t-test, p < 0.001). Eye area and neuromast hair cell analysis were performed with 10 embryos for each group. Graphs show the mean and s.e.m. Replication of the experiment produced similar results.

One of the endpoints for nucleotide synthesis is to provide energy for cell metabolism. As production of the primary unit of energy in the cell, ATP, relies on the nucleotide biosynthesis pathway, we hypothesized many of the phenotypes could be a direct result of energy starvation in the cells. To examine whether *prps1* mutant phenotypes could result from an energy deficiency, we reduced ATP production in wild-type embryos using antimycin A, an inhibitor of the mitochondrial electron transport chain^21^. Antimycin A treatment caused a reduction in eye size and iridophores (Fig. 7A–C), as well as a reduction in neuromast hair cell numbers (Fig. 7D) similar to the *prps1* mutants. The severity of these phenotypes increased with higher concentrations of antimycin A similar to the increased phenotype severity seen from an increased number of mutant alleles.

### Increased sensitivity of *prps1a;prps1b* double mutants to inhibitors

To investigate whether *prps1* mutant phenotypes are caused by reduced nucleotide and/or energy levels, we applied an intermediate concentration of leflunomide and antimycin A to the *prps1a;prps1b* double mutant embryos generated from a pairwise in-cross of adult fish carrying *prps1a* heterozygous and *prps1b* homozygous mutations. A total of 76 embryos were used for leflunomide treatment at 1 μM. The treatment led to three distinct classes of embryos with the morphological difference mainly in the eye size and pigmentation (Suppl. Fig. 8A–C). Genotyping of these three classes of embryos revealed there was an enrichment of the double mutant in the most severely affected embryos. Among the 18 double mutants identified from genotyping, 11 out of 18 showed phenotype as in C, 7 out of 18 showed phenotype as in B, and none showed phenotype as in A. It is worth noting that the phenotypes of 1 μM leflunomide treated *prps1a;prps1b* double mutants (Suppl. Fig. 8C) resembled the phenotypes of 2 μM leflunomide-treated wild-type embryos (Suppl. Fig. 6A). These data indicate that leflunomide treatment can separate *prps1a;prps1b* double embryos from their siblings based on sensitivity to inhibition.

Antimycin A treatment was performed by exposing 32 similarly obtained embryos to 0.5 ng/ml of antimycin A. The treatment produced two classes of embryos. One class displayed a slight reduction in eye size (Suppl. Fig. 8D), all 11 embryos genotyped from this class were wild type or heterozygotic for the *prps1a* mutation. The other class of the embryos displayed a dramatic reduction of eye size together with other phenotypes including heart edema, short body and necrotic tail tissue (Suppl. Fig. 8E). All 8 embryos genotyped from this class were homozygotes for the *prps1a* mutation. The data from antimcyin A treatment demonstrate that the *prps1a;prps1b* double mutants are hypersensitive to the inhibitor compared to their control siblings. We infer this is because of a reduction in ATP availability.

### SAM does not rescue *prps1a* and *prps1a:prps1b* mutant phenotypes

A recent open-label clinical trial of dietary supplementation of S-adenosylmethionine (SAM) appears to have alleviated some of the symptoms in two Australian brothers with Arts syndrome^22^. To test the effect of SAM in our *prps1* model, we provided SAM in the embryo medium or by yolk injection to *prps1a* embryos generated by an in-cross of *prps1a* heterozygous parents, or *prps1a;prps1b* double mutant embryos generated from an in-cross of *prps1a* heterozygous and *prps1b* homozygous parents. We found no significant rescue on either *prps1a* or *prps1a;prps1b* mutant phenotypes, even at the highest safety concentration of 0.2 mM (data not shown), suggesting the symptom improvement in the Arts patients could be attributed to other causes.

### *prps1a;prps1b* double mutants show severely reduced levels of PRPP and other metabolic changes

To define the metabolic disruptions caused by the *prps1a;prps1b* double mutants, we performed ionic metabolite measurements and calculated the ratio between the *prps1a;prps1b* double mutants and their genetically related wild-type control embryos (Table 1). A dramatic reduction was detected in the level of PRPP, the direct product of Prps1, confirming the severe reduction of Prps1 activity in the double mutants. The metabolite measurement also revealed significant reductions in uric acid and inosine 5′-monophosphate (IMP), two metabolites linked to the PRPS1-associated nucleotide synthesis pathway. Consistent with the results from antimycin A inhibition, we detected moderate reductions in both GTP and ATP. Supporting the failed rescue of the double mutant phenotypes with SAM, we found SAM level was not reduced in the double mutant. The levels of purine, pyrimidine and pyridine could not be measured in either control or mutant samples with the methods used.

## Discussion

In this study, we created zebrafish mutants for *prps1a*, *prps1b*, and the double mutant *prps1a;prps1b*. We found the *prps1a* mutants and *prps1a;prps1b* double mutants showed similar phenotypes with an increasing degree of severity in the double homozygous mutant over either single homozygous mutant. The *prps1b* mutation does not cause any obvious morphological phenotypes, however when placed in the *prps1a* mutant background, the resulting *prps1a:prps1b* double mutants had more severe phenotypes than the *prps1a* mutation and the majority of the fish did not survive to adulthood. These phenotype observations, along with their distinct expression patterns, demonstrated that *prps1a* is the dominant paralog in zebrafish, while *prps1b* offers additional expression that is sufficient for survival in the absence of *prps1a*.

The different combinations of zebrafish mutants represent models for different levels of Prps1 activity reduction. The *prps1a* mutant can act as a model for a moderate reduction of Prps1 activity. The *prps1a* mutant has measurable defects in the eyes and hair cells early in development, but can grow to be fully functional adults, paralleling the relatively mild optic atrophy and hearing impairment observed in the patients with X-linked Charcot-Marie-Tooth disease-5 (CMTX5) who have a normal life span. The *prps1a:prps1b* double mutant represents a model for a more severe reduction of Prps1 activity. The larger reduction in Prps1 activity in the double mutants resulted in worsened morphological phenotypes and a shortened life span, most often embryonically lethal. This resembles the features of more severe human *PRPS1* mutations such as Arts Syndrome that have increased phenotypic severity and premature death. The abnormal axon projection of primary motor neurons and the reduction of leukocytes in the *prps1a;prps1b* double mutant are also consistent with the delayed motor development and recurrent infections observed in Arts syndrome. Moreover, the dysmorphic craniofacial features and short body length observed in the *prps1a;prps1b* double mutants have also been reported in human patients with the most severe forms of PRPS1 deficiency^6^. The observation that mutant embryos were hypersensitive to the chemical inhibitors helps support the model that the observed variation in phenotypes are directly linked to the relative level of Prps1 activity, and inhibition of the electron transport chain showed that potentially the primary problem in the mutant fish is energy starvation.

We found most of the morphological phenotypes in the mutants arose in the tissues with the highest relative *prps1a* and *prps1b* expression. Both *prps1a* and *prps1b* are enriched in the embryonic nervous system, particularly the brain and retina. The notochord also showed an elevated level of expression. A higher level of *prps1a* expression in the ciliary marginal zone (i.e. the growth zone) of eyes (Fig. 1) suggests an enhanced need for *prps1a* activity in actively dividing cells, as demonstrated by the small eye phenotype observed in the *prps1a* mutants. That the eye phenotype was connected to the *prps1a* allele is notable because *prps1b* was not detected in the ciliary marginal zone, the area of active growth in the retina. This lends credence to the idea that the cell populations that are dividing fastest or have increased energy needs are most sensitive to the loss of Prps1 activity.

Consistent with our data, studies have shown that mutations in genes downstream of *PRPS1* in the nucleotide synthesis pathway cause similar phenotypes. For examples, small-eye and reduced pigmentation phenotypes were observed in zebrafish mutations for *gart* and *paics*^14^. Abnormal retinal axon projection was reported in *Drosophila* mutations for *GMP synthase* and *impdh*^15^.

Hearing impairment was reported in many of the *PRPS1*-related human patients, suggesting PRPS1 plays an important role in hearing function or development. We found both zebrafish *prps1a* and *prps1b* were enriched in the zebrafish inner ear (Fig. 1H and data not shown), and the *prps1a;prps1b* double mutants had a significant reduction in the number of inner ear hair cells (Fig. 4J–L). In addition to the reduction in the inner ear hair cells, the *prps1a;prps1b* double mutants also had significantly reduced neuromast hair cells (Fig. 4I). A comparable level of reduction of neuromast hair cells is observed in the *prps1a* mutant, suggesting even a partial reduction of Prps1 activity is sufficient to reduce hair cell numbers. Consistent with our observations, mouse *Prps1* expression is enriched in the inner ear hair cells^11^. In humans, a weak reduction of PRPS1 activity, as seen in DFNX1 patients, causes hearing impairment with no additional symptoms at an early age^11^, suggesting hearing is particularly sensitive to functional reduction of *PRPS1*.

In Arts syndrome, a disease with a severe reduction of PRPS1 activity, recurrent infection is characteristic and often causes early death^23^. In zebrafish, both *prps1a* and *prps1b* expression are enriched in the caudal hematopoietic tissue (Fig. 1E, Suppl. Fig. 1E), and severe reduction of *prps1* activity in the *prp1a;prps1b* double mutants caused a significant decrease of both neutrophils and macrophages (Fig. 6). Consistent with our data, other studies have also shown reduction in PRPS1-mediated nucleotide synthesis causes infections associated with leukocyte deficiencies in humans. For examples, the patients with mutations in the pyrimidine synthesis gene *UMPS and* the purine synthesis gene *PNP* have T cell deficiencies^24^,^25^, and patients with mutations in the purine synthesis gene *ADA1* have deficiencies in both T cells and B cells^26^. Finally, mutations in *ADA2*, another gene involved in purine metabolism, caused mild lymphopenia in humans and reduced neutrophil development in the zebrafish^27^,^28^. Taken together, these data strongly suggest PRPS1-related nucleotide synthesis is an important factor in leukocyte homeostasis and infection prevention.

One striking feature of the zebrafish *prps1* mutants is that most of the phenotypes recover over time. One example is the recovery of small-eye phenotype. Small eye and pigmentation phenotypes were observed in the *prps1a* mutant embryos (Fig. 3, Suppl. Fig. 2), but not in the adults. Melanocyte reduction was observed in *prps1a;prps1b* double mutants at 1 dpf (Suppl. Fig. 4A), but not later. The retinal pigment epithelium was thinner in *prps1a* mutants at 3 dpf, but was normal by 6 dpf (Suppl. Fig. 2D). Reduced neuromast hair cells were detected in both *prps1a* (Fig. 3N) and *prps1a;prps1b* double mutants at 3 dpf (Fig. 4I), but not at 5 dpf (Suppl. Fig. 2E, data not shown). The double mutants have reduced leukocytes during early embryo development (Fig. 6). However, the reduction is not detected in the few surviving double mutant adults (Suppl. Fig. 4F–G).

Because zebrafish continue to grow throughout their lifetime, it is possible that *prps1* mutants recover because there is not a “cutoff” point after which development will not continue, In the case of human *PRPS1* patients, this simply may not be the case. There are some fundamental ramifications that result from the differences between humans and zebrafish regarding tissue development and homeostasis. For example in hair cell development, humans grow their inner ear hair cells during early embryonic development and then there is no new hair cell growth after birth^29^. In contrast, zebrafish grow new hair cells throughout their lifetime^30^ and have the capacity to regenerate hearing after damage^31^. Reduced hair cells in zebrafish *prps1a* mutant and *prps1a;prps1b* double mutant suggest that human patients with *PRPS1* mutations could possibly possess fewer hair cells at birth, as evidenced by the finding of hearing loss in a patient of 5 months of age^12^ and are thus more prone to measurable hearing loss. Consistently, humans have no ability to repair the retinal neurons that are degenerated from genetic and environmental causes, while zebrafish can restore retinal damage very efficiently^32^,^33^. Thus the early onset of phenotypic manifestations of the *PRPS1* patients could suggest a developmental defect and environmental sensitivity that cannot be repaired or replaced after birth.

Analysis of the *prps1* retinal phenotype suggests that *prps1* mutant has an altered cell cycle similar to that seen in *gart* and *paics* mutants^14^ (Fig. 3E–K). The extended cell cycle very likely results in the slow growth phenotypes that recover over time in the zebrafish mutants. Partial phenocopy of the *prps1* mutant phenotypes by pharmacological inhibitions of nucleotide synthesis or ATP production (Fig. 7, Suppl. Figs 6–8) and reduction of Prps1-pathway intermediates and energy molecules revealed by metabolite analysis (Table 1) together demonstrate the phenotypes are primarily from limited nucleotide and ATP availability, supporting nucleotide synthesis impacting the cell cycle as previously reported^18^,^34^,^35^. Consistent with our data, other studies have shown an association between PRPS1 function and cell cycle progression. Targeting PRPS1 by MicroRNA-124 reduces the proliferation of human colorectal cancer cells^36^. Zebrafish mutations in *gart* and *paics*, two genes downstream of PRPS1 in nucleotide synthesis pathway, have similar retinal phenotypes that are also caused by a prolonged S phase of proliferation and slower cell division rate due to deficient ATP production^14^. In addition, ATP released from the retinal pigment epithelium regulates eye growth^37^,^38^. Furthermore, another *Prps* gene, *Prps2*, directly promotes cancer cell proliferation in mice^39^. Since the *prps2* and *prps3* genes have not been identified in zebrafish, we could not examine whether there is cross-regulation between the different *prps* genes. Our data show the *prps1*-associated phenotypes predominately localize in the tissues enriched with *prps1* expression, it is easy to envision that lack of *prps1* in these tissues reduces local production of nucleotides and the energy molecule ATP, ultimately affecting DNA replication and cell cycle progression and slowing down tissue development in a seemingly specific fashion.

We also observed some neurological defects in *prps1* mutants, including abnormal axon branching of primary motor neurons (Fig. 5A–E) and enlarged afferent innervation of neuromast hair cells (Fig. 5F–H), features not associated with developmental delay. Understanding these anomalies in the context of loss of *prps1* activity will require further study but may suggest a clue towards the neuropathology observed in the patients.

In summary, we took advantage of a gene duplication in zebrafish to generate mutant models for different levels of Prps1 activity reduction that imitate the functional and phenotypic spectrum of the human patients and showed the key pathologies are linked to nucleotide and energy production. These models could be used for in-depth investigation of the role of *PRPS1* in development and the pathology of *PRPS1*-associated human diseases, and potentially for identifying new therapies for patients. The generation of “humanized” *PRPS1* mutation models could shed further light on the the nature of the disease for individual patients carrying specific PRPS1 mutations.

## Methods

### Ethics statement

All animal work was in compliance with NHGRI IACUC approved protocol G-01-3 assigned to SMB. All procedures were also in compliance with the NRC “Guide for the Care and Use of Laboratory Animals.”

### Zebrafish husbandry

All experiments were approved by the NHGRI Animal Care and Use Committee (protocol # G-10-3) and all experiments were performed in accordance with relevant guidelines and regulations. Zebrafish embryos were obtained from natural crosses and tightly staged according to Kimmel^40^. All experiments were performed on a mix of male and female fish, and all mutants were derived in the TAB-5 background. The *prps1a*^*la015591*^ mutation was generated by retroviral insertional mutagenesis^16^. The *prps1b*^*hg19*^ mutation was generated by microinjection of a mixture of synthetic mRNAs encoding two zinc fingers fused to the fokD1 nuclease. For RT-PCR and qRT-PCR analysis, 20 embryos for 1 hpf, 5 dpf and 3 hpf, or 3 juvenile fish at 40 dpf were used for total RNA extraction, cDNA synthesis and then PCR or qPCR analysis. Primers used: *prps1a* forward [1F]: AGG AGA GAG TGT CCG TGG AG; *prps1a* reverse [5R]: ACC TCA TTG GCC TTC TTC CT; *prps1b* forward: TCC ATT TTG CTT GTG CAC TC; *prps1b* reverse, CAG CAT CGG GAG ACA CAA T; *β-actin* forward, TTG TGA CCA ACT GGG ATG AC; *β-actin* reverse, AGC ACT TCC TGT GAA CGA TG. qRT-PCR were done with Power SYBR green PCR master mix (Applied Biosystems, Cat# 4367659), and the data were analyzed as a percentage compared to the level of *prps1a* or *prps1b* at 1 h post fertilization (with the expression levels in the embryos at 1 hpf adjusted to 100%). For whole mount *in situ* hybridization, specific probes were designed for *prps1a* and *prps1b*. Primers used for *prps1a* probe synthesis: *prps1a* probe-F: ATT AAC CCT CAC TAA AGG GAC TTG AAA CGG AGG AGA TAC CC. *prps1a* probe-R: TAA TAC GAC TCA CTA TAG GGA GAG ATG CGA GCT ACC GCT AAA T; The resulting probe bound to the 5′UTR of the *prps1a* gene. Primers used for *prps1b* probe synthesis: *prps1b* probe-F: ATT AAC CCT CAC TAA AGG GAG CCA GCA CAT GCT TAT TTG TAG; *prps1b* probe-R: TAA TAC GAC TCA CTA TAG GGA GAC AGA CTG GCA TAA TGT AAA CAA AGG. The resulting probe bound to the 3′UTR of the *prps1b* gene. For measuring the knockdown efficiency of the mutations, additional primers were used, including: *prps1a* forward: CAG GCC CTC TGC TTA ACA TC; *prps1a* reverse: GGT CTT TCC AGT ACT CTT GGA TCT C.

### prps1a CRISPR mutant generation

The *prps1a* CRISPR mutants were generated by CRISPR/Cas9 gene targeting. The single guide RNA’s (sgRNA) were designed using CRISPRscan^41^, targeting exon 1 of the gene. The sequence of the guide RNA is taatacgactcactata GGGACTCGAGCTGGGGAAAG gttttagagctagaaatagc. Lower cases at 5′ and 3′ of the sequence indicate T7 promoter and partial guide RNA sequences. Upper case letters indicate the target genomic DNA sequence in exon 1 located at chr5: 24805721-24805740. Cas9 mRNA and sgRNA were synthesized as previously described^42^. 150 pg of Cas9 mRNA was either injected alone (for control) or co-injected with 50 pg of *prps1a* sgRNA (for knockdown of *prps1a*) into the cells of wild-type embryos at 1-cell stage. Phenotypes were analyzed at 3 dpf using 10 representative embryos from each group. The analyzed embryos were afterwards genotyped using CRISPR-STAT as reported^43^, with primers prps1a M13-F (TGT AAA ACG ACG GCC AGT CCC GAC ACT ATA GGC ACC AC) and prps1a-PIG-R (GTG TCT TCG GTT ACC CTA GCC AAA ACA).

### Histology

For mRNA expression analysis, whole mount *in situ*-stained 5-day-old embryos were embedded in paraffin, transversely sectioned at a thickness of 5 micron (μm), and then counterstained by light nuclear fast red. For retinal lamination analysis, mutants and control siblings at the indicated stages were sequentially infiltrated at 4 °C with 15% and 30% sucrose (in 1x phostphate buffer saline, PBS). Embryos were embedded in optimal cutting temperature compound (OCT, Tissue-Tek) and transversely section at 10 μm. Samples were air-dried at 25 °C for one hour and stained with hematoxylin and eosin (H&E). For retinal cell proliferation analysis, mutant and control siblings at 3 dpf were transversely sectioned at 10 μm and placed on Colorfrost Plus slides (Fisher). Sections were rehydrated with PBTD (1xPBS, 0.1%Tween, 1% DMSO), blocked with PBTD with 5% goat serum for 2 hours. Primary antibody anti-phospho Histone H3 (Ser10) (Millipore; 06-570; Lot #: JBC1903648) was diluted 1:100, applied and the samples incubated overnight at 4 °C. ProLong Gold anti-fade (Invitrogen) containing DAPI as a counterstain, was used to mount samples.

### Hair cell development analysis

The mutants and control siblings at the indicated stages were used for hair cell quantification. For neuromast hair cells, the embryos were stained with Yopro-1 (Molecular Probe, Cat# Y3603) as described previously^44^. The stained embryos were orientated to a lateral view in 96-well plate format for imaging and counting using a fluorescent microscope. Hair cells in the P1–P4 neuromasts were counted for each embryo. The hair cell number shown in the graphs was an average of hair cell numbers from approximately 10 embryos. For inner ear hair cell analysis, the embryos were stained with a combination of two monoclonal antibodies specific to hair cells, myosin-VIIa (Developmental Studies Hybridoma Bank, MYO7A 138-1, 1 μg/ml) and hair cell soma-1 (Developmental Studies Hybridoma Bank, HCS-1, 1 μg/ml), and a secondary antibody conjugated with Alexa 488 (Invitrogen, A11001, 4 μg/ml). The stained embryos were dissected and the heads were mounted in 1% low melting agarose for a lateral view. The imaging and counting were done using a confocal microscope. Approximately 10 embryos were used for each data point.

### Primary motor neuron analysis

The control and the double mutant embryos at 32 hpf and 48 hpf were used for primary motor neuron staining. Primary motor neurons were labeled with anti-znp-1 monoclonal antibody (Developmental Studies Hybridoma Bank, znp-1, 1 μg/ml) and a secondary antibody conjugated with Alexa 488 (Invitrogen, A11001, 4 μg/ml). The stained embryos were mounted in 1% low melting agarose for a lateral view and imaged using a fluorescent microscope. Images were framed on the five axons immediately anterior to the end of yolk extension.

### Neuromast hair cell innervation analysis

*prps1a;prps1b* double mutants and control siblings at 6 dpf, together with wild type embryos at 4 dpf, were stained with antibodies against RibeyeA (as described by Sheets *et al*.)^45^, Membrane Associated Guanylate Kinase (MAGUK) (NeuroMab, AB# 28/86), and Calretinin (Swant, AB# 6B3) following manufacture’s instructions. Primary antibodies were diluted at [1:1,000] (RibeyeA and Calretinin) or [1:500] (MAGUK). Alexa Fluor (anti-mouse IgG2a 488, anti-mouse IgG1 546 and anti-rabbit 647) secondary antibodies were diluted at [1:1,000]. The stained embryos were mounted on slides using ProLong Gold Antifade Mountant (Molecular Probes) and stored at 4 °C in the dark. For image analysis, Z-stacked images were acquired every 0.48 μm using a Zeiss LSM780 confocal microscope using 488, 561 and 647 nm laser lines. Image J was used for processing and quantification of all images. Threshold images were produced for pre-synaptic ribbon and post-synaptic MAGUK puncta quantification. Background subtraction was used for MAGUK quantification. The minimum particle size for inclusion in each data set was 0.07 μm^2^ per pre or post-synaptic punctum. To quantify afferent neuron innervation, threshold images were created and a bounding rectangle was formed around the area containing Calretinin stain. The area of each rectangle enclosing the stain was quantified. A minimum of 10 neuromasts was quantified for each data set. Statistical significance was defined by the Tukey’s multiple comparisons test.

### Chemical treatment

Three chemicals were used to probe the mechanism of *prps1*-associated phenotypes, including leflunomide (Sigma, Cat# L-5025), mycophenolate mofetil (Sigma, Cat# SML0284) and antimycin A (Sigma, Cat# A8674). For inhibiting nucleotide synthesis, wild-type embryos were treated without or with different concentrations of leflunomide or mycophenolate mofetil from 6 hours post fertilization (hpf) until 3 days post fertilization (dpf). For antimycin A treatment, wild-type embryos were treated without or with different concentrations of antimycin A from 24 hpf until 3 dpf. Embryonic phenotypes were analyzed at 3 dpf. For rescuing *prps1* mutant phenotypes, SAM (Sigma, Cat# A7007) was either supplemented in the medium at various concentrations ranging from 0–0.2 mM from 6 hpf until 5 dpf, or injected 0 or 15 ng per embryo in embryonic yolk at 19 hpf, to the embryos born from incross of *prps1a* heterozygotic and *prps1b* homozygotic parents. The analysis on rescue was examined at 3 dpf. Medium supplement of all above 4 chemicals were conducted in 1x Holtfreter’s buffer with fresh compounds replenished every other day. The treated embryos were placed on a shaker that rotates at 40 rounds per minute to improve chemical distribution. The vehicle treated embryos were processed in the same way as those exposed to chemical inhibitors to ensure the accuracy of the results.

### Hematopoietic blood cell lineage analysis

Three control and three double mutant zebrafish at 4 months were used for blood cell lineage analysis. These adult fish were euthanized in an ice bath and then decapitated for blood collection. The blood was immediately smeared on glass slides. Wright Giemsa staining was used to distinguish different types of blood cells^46^. Areas with well-separated blood cells in the slides were imaged and used for cell counting. Cell counts were performed on erythrocytes, myeloid cells that include monocytes/macrophages and neutrophils, as well as hematopoetic progenitors. For data analysis, counts of myeloid cells and progenitor cells for each fish were normalized to the erythrocyte counts.

### Metabolite measurement

The *prps1a;prps1b* double mutant embryos generated from an incross of *prps1a* heterozygous and *prps1b* homozygous adult fish and genetically related wild-type controls were used for ionic metabolite measurement performed by Human Metabolome Technologies Inc., following the standard protocol for capillary electrophoresis time-of-flight mass spectrometry (CE-TOPMS). Briefly, three groups of *prps1a;prps1b* double mutants or controls at 4 dpf (approximately 50 mg of tissues for each group) were collected and completely depleted of embryo medium. The samples were homogenized with 4500 μl of 50% acetonitrile in water (v/v) containing internal standards. The filtrated supernatants were concentrated and re-suspended in 50 μl of ultrapure water before performing CE-TOPMS. Two dilution conditions were tested. The metabolites were separated and identified using fused silica capillaries under both anion and cation modes. The data were analyzed by automatic peak integration software. The ratio of change in the double mutants was calculated by dividing the peak area of the mutants by that of the controls.

### Statistical analysis

The statistics were done using a student *t-test* (two tailed), except when otherwise indicated. A difference was considered significant when the p value was less than 0.05. Bar graphs show the mean value and the standard error of the mean (s.e.m.), except as otherwise indicated. All the experiments shown were performed at least two times and produced consistent results.

**Ethical Approval:**  All animal experiments were approved by the Animal Care and Use Committee of the National Human Genome Research Institute, protocol number G-03-1, and all institutional and national guidelines were adhered to.

## Additional Information

**How to cite this article**: Pei, W. *et al*. Additive reductions in zebrafish *PRPS1* activity result in a spectrum of deficiencies modeling several human *PRPS1*-associated diseases. *Sci. Rep*. **6**, 29946; doi: 10.1038/srep29946 (2016).

## Supplementary Material

Supplementary Information

Supplementary Information

## Figures and Tables

**Table 1:** Metabolite analysis of the *prps1a;prps1b* double mutants.

Metabolites	prps1ab^*−/−*^/WT		
	Ratio	s.e.m.	*p*-value
PRPP	<0.01	n.a.	0*
Uric acid	0.57	0.007	0.0003*
IMP	0.82	0.019	0.0114*
GTP	0.74	0.054	0.0411*
ATP	0.78	0.025	0.0123*
SAM	1.25	0.064	0.0559

The ratio of change between the double mutants and the wild-type controls for 6 PRPS1 pathway-related metabolites. The ratio is calculated by dividing the peak area of the mutants by that of the control siblings. s.e.m. stands for standard error of the mean. P value is calculated by two tailed student t–test. Asterisks indicate a significant difference between the control and the double mutant. Since PRPP is detected in the control but not in the mutants, the ratio is labeled as <0.01, s.e.m. as not applicable (n.a.).
