# Supplementary material for: Additive reductions in zebrafish PRPS1 activity result in a spectrum of deficiencies modeling several human PRPS1-associated diseases
Source: Sci Rep. 2016 Jul 18;6:29946. doi: 10.1038/srep29946 (PMC4947902; doi:10.1038/srep29946)
Supplement: Supplementary Information [file srep29946-s1.pdf]

Additive reductions in zebrafish *PRPS1* activity result in a spectrum of deficiencies modeling several human *PRPS1*-associated diseases

Wuhong Pei[1], Lisha Xu[1], Gaurav K. Varshney[1], Blake Carrington[1], Kevin Bishop[1], MaryPat Jones[2], Sunny C. Huang[1], Jennifer Idol[1], Pamela R. Pretorius[3], Alisha Beirl[4], Lisa A. Schimmenti[5], Katie S. Kindt[4], Raman Sood[1], Shawn M. Burgess[1]

[1] Translational and Functional Genomics Branch, National Human Genome Research Institute, Bethesda, MD 20892, USA.

[2] Cancer Genetics and Comparative Genomics Branch, National Human Genome Research Institute, Bethesda, MD 20892, USA.

[3] Department of Biology, Hanover College, Hanover, IN, 47243, USA.

[4] Section on Sensory Cell Development and Function, National Institute on Deafness and Other Communication Disorders, Bethesda, MD 20892, USA.

[5] Mayo Clinic, Rochester, MN 55905, USA.

Suppl. Fig. 1

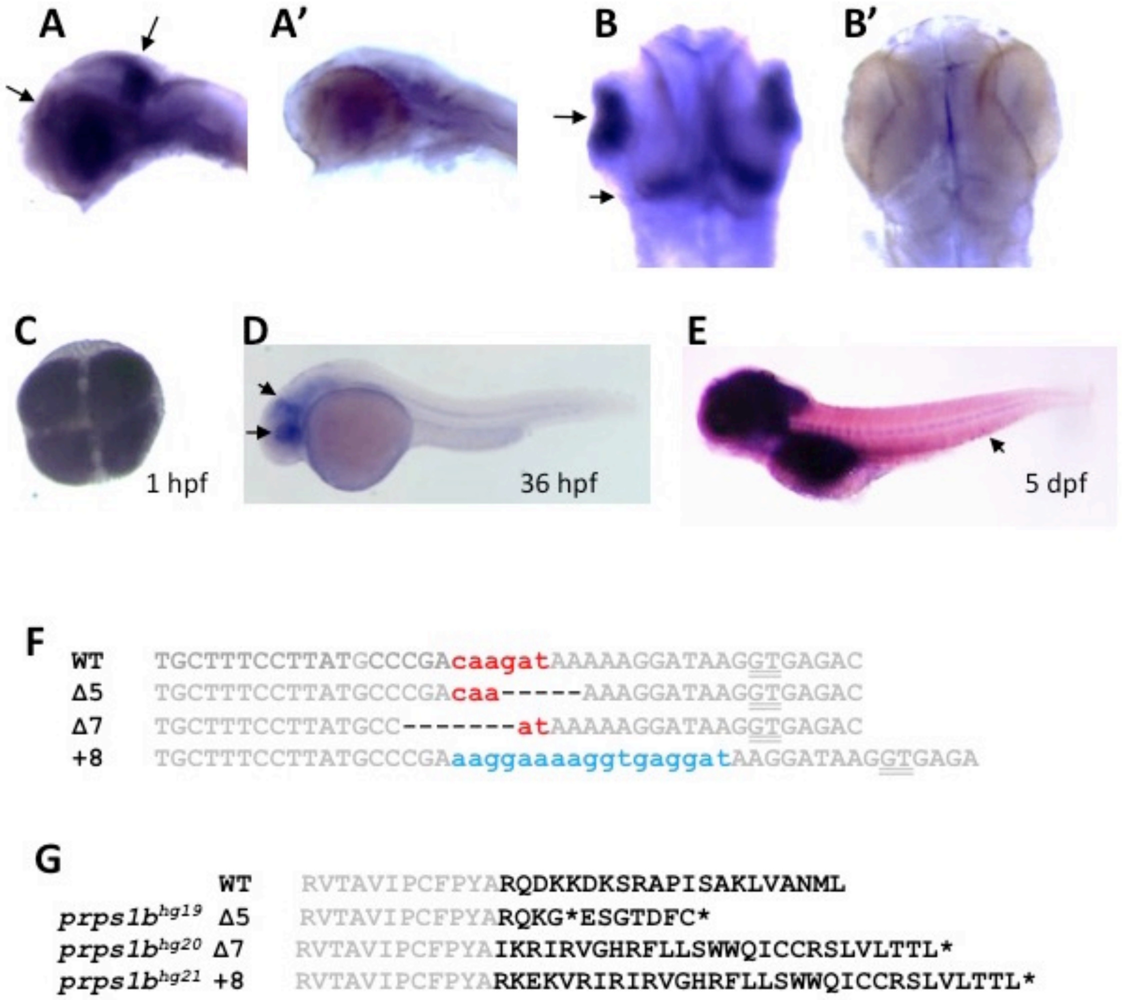

Suppl. Fig. 1. *prps1* gene expression and *prps1b* mutations.

(A-B) Flat mount images show the specific expression of *prps1a* in the retina and mid-brain hindbrain boundary. Wild-type embryos at 36 hpf were used for whole-mount in situ hybridization analysis with the antisense (A,B) and sense (A’,B”) probes. A and A’, lateral views. B and B’, dorsal views. Black arrows in A and B point to the staining in the retina and brain. (C-E) *prps1b* expression in the wild-type embryos at the 4-cell stage (C), 36 hpf (D) and 5 dpf (E), detected by whole-mount in situ hybridization. Black arrows in D point to the enrichment in eye and tectum.

Arrow in E points to the enrichment in caudal hematopoietic tissue. Images in C and D are processed for the same amount of time. Image in E is from an extended staining to show the expression in the caudal hematopoietic tissue. (F) DNA sequence alignment of the three *prps1b* frame-shift mutations. The wild-type (WT) DNA sequence is from Vega transcript OTTDART00000037699. ZFN target sites are shown in grey. Spacer sequence is shown in red. Deletions are shown as dashes (-). Insertions are shown in blue. Splice site GT is underlined. (G) Protein sequence alignment of the three *prps1b* frame-shift mutations. The WT protein sequence is from UniProt: Q08CA5_DANRE. Conserved residues are shown in grey. Premature stop codons are shown as asterisks (*). There are presumably 2, 23 and 9 extra amino acid residues introduced prior to the premature stop codon for the 5 bp deletion, 7 bp deletion, and 8 bp insertion mutations, respectively. For the 5 bp deletion mutation, there is a second premature stop codon shortly after the first one.

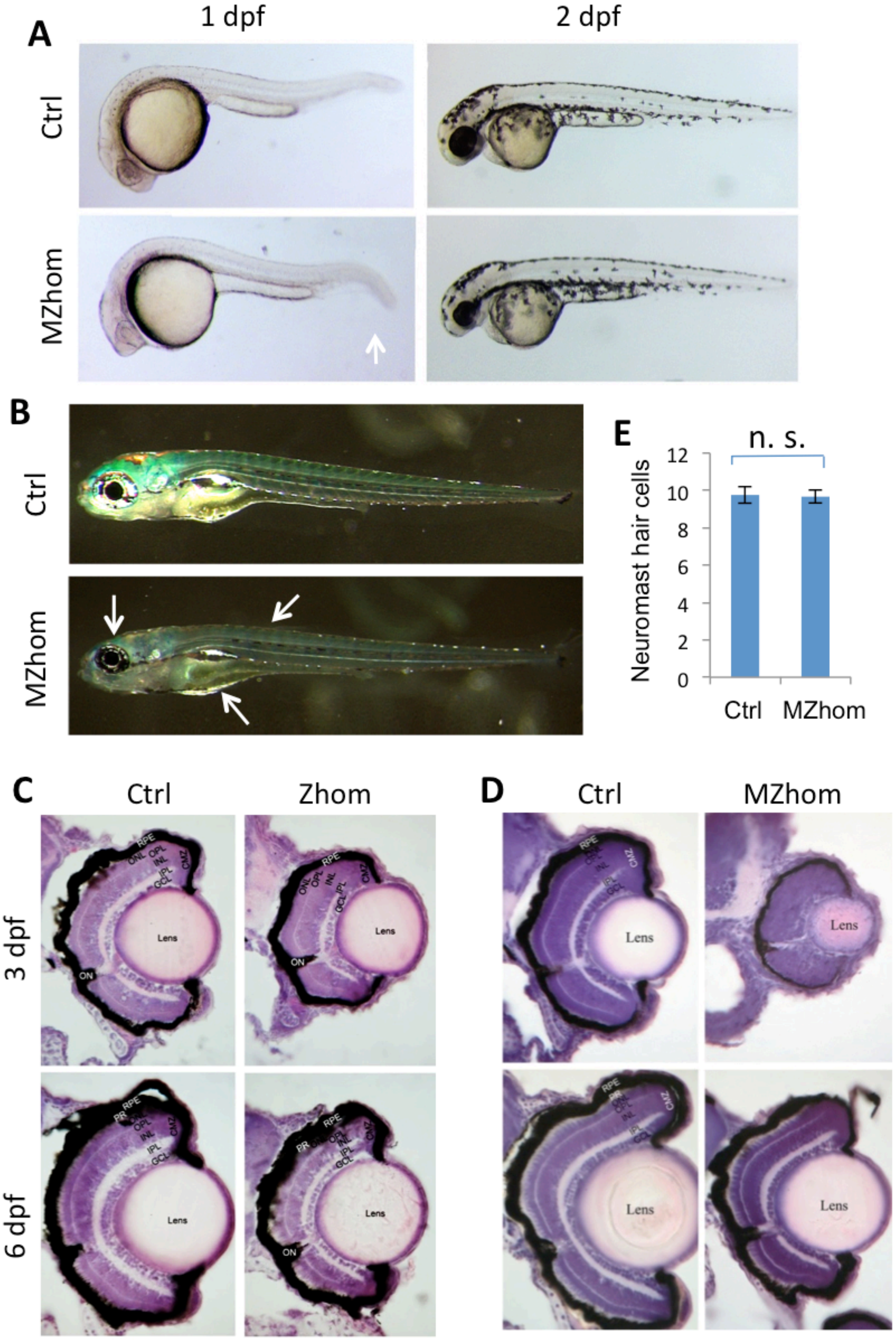
A
1 dpf
2 dpf
Ctrl
MZhom
B
Ctrl
MZhom
E
n. s.
Neuromast hair cells
12
10
8
6
4
2
0
Ctrl
MZhom
C
Ctrl
Zhom
3 dpf
6 dpf
D
Ctrl
MZhom
Lens

Suppl. Fig. 2. *prps1a* mutant phenotypes.

(A) The smaller eye phenotype in *prps1a* MZhom embryos at 1 dpf and 2 dpf. White arrow indicates easily visible reduction in size. (B) Reduced iridophores in *prps1a* MZhom at 5 dpf. White arrows show where the reduction of iridophores in the head, trunk and yolk are most easily seen. (C) Retinal morphology of *prps1a* Zhom embryos at 3 dpf and 6 dpf. (D) Retinal morphology of *prps1a* MZhom embryos at 3 dpf and 6 dpf. CMZ, ciliary marginal zone. GCL, ganglion cell layer. IPL, inner plexiform layer. INL, inner nuclear layer. OPL, outer plexiform layer. ONL, outer nuclear layer. PR, photoreceptor. RPE, retina pigment epithelium. Eyes are most reduced at 3 dpf, but begin to recover by day 6. (E) *prps1a* homozygous mutants have a normal number of neuromast hair cells at 5 dpf. The difference between the control and mutants is not significant (n=10, p=0.87).

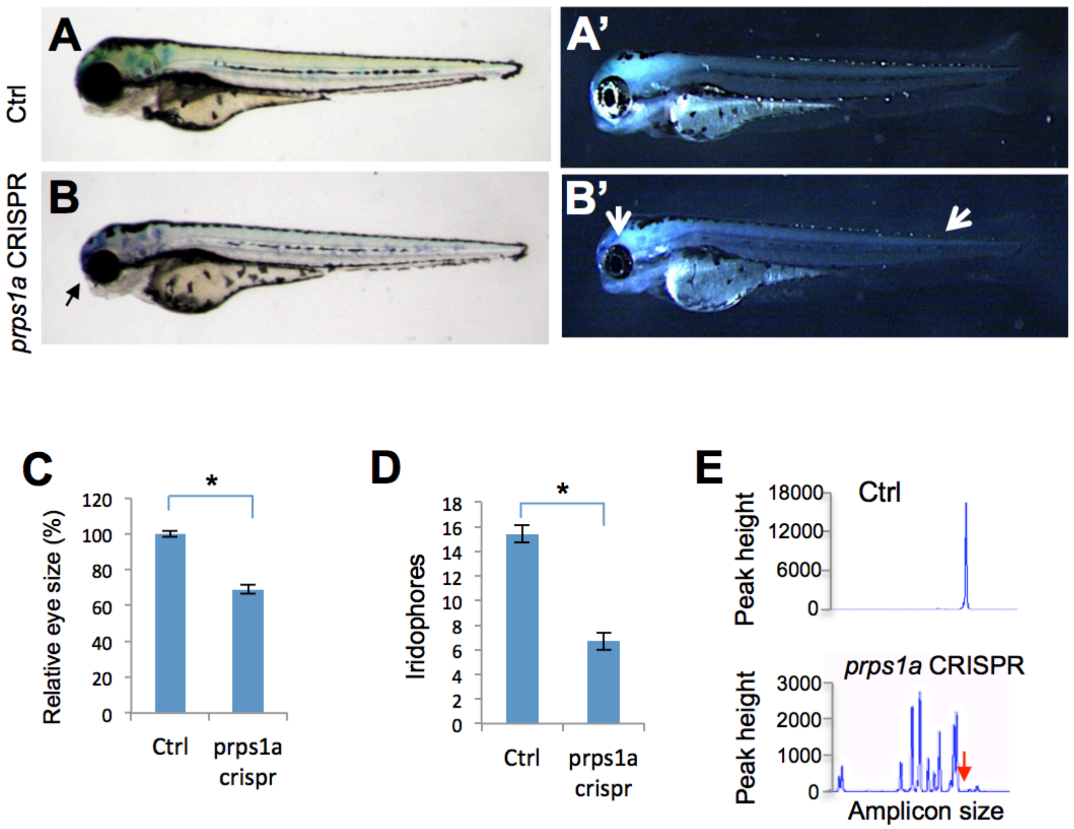

Suppl. Fig. 3. ***prps1a*** **CRISPR mutant phenotypes.**

(A-B) Small eye phenotype in *prps1a* CRISPR mutants at 3 dpf. Black arrow points to the small eye in the mutant. (A'-B') Iridophore phenotype in *prps1a* CRISPR mutants at 3 dpf. Two white arrows point to the reduced iridophores in the eye and dorsal trunk of the mutant. (C-D) Quantification of the reduction in eye size (C) and iridophores (D). The eye areas were calculated using image J. The numbers of iridophores were obtained by counting the iridophores in the dorsal and ventral side of the trunk area above the yolk extension. The reduction was significant in the eye size (n=10, p<0.001) and iridophores (n=10, p<0.001). (E) Representative fluorescent PCR plots showing the peaks indicating efficient somatic mutation. The representative plots are from Control # 4 and *prps1a* CRISPR #6 embryos (details in Suppl. Table 1). The X-axis represents the size of the target amplicon. The Y-axis shows the peak height for each amplicon

size. The red arrow points to the position of the WT peak. All peaks shown are within 50 base pair of the WT peak. Ctrl is uninjected sibling fish.

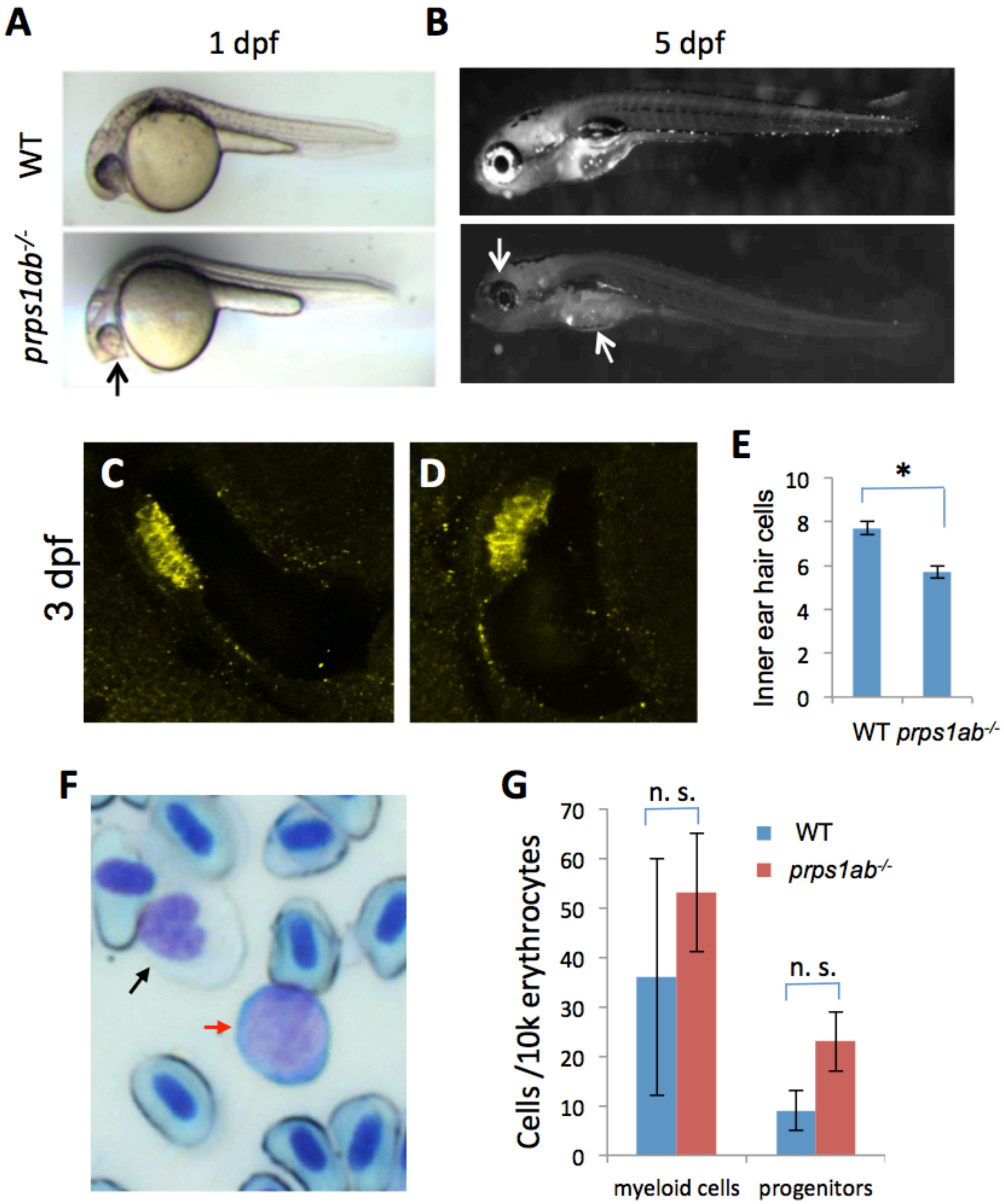

Suppl. Fig. 4. *prps1a;prps1b* double mutant phenotypes.

(A) Reduced melanocyte pigmentation in the double mutant at 1 dpf. *prps1ab*$^{-/-}$ indicates *prps1a;prps1b* double mutant. Black arrow points to the small and poorly-pigmented eye of the double mutant. (B) Reduced iridophores in the double mutants at 5 dpf. White arrows indicate the reduced iridophores in the eye and yolk of the double mutant. (C-D) Inner ear hair cells in 3 dpf

embryos were stained with a combination of myosin-VIIa and hair cell soma-1 antibodies. 11 embryos per group were used for inner ear hair cell analysis. Representative images are shown. (E) Quantification of the reduction of inner ear hair cells. The reduction in the double mutants is significant (n=11, p<0.003). (F) Blood cell lineage analysis by Wright Giemsa staining. Black arrow indicates a myeloid cell and red arrow indicates a progenitor cell. Other cells are erythrocytes. (G) Quantification of myeloid cells and progenitors in the 3 control adults and 3 double mutant adults at 4 months. The differences were not significant (n. s.).

Suppl. Fig. 5

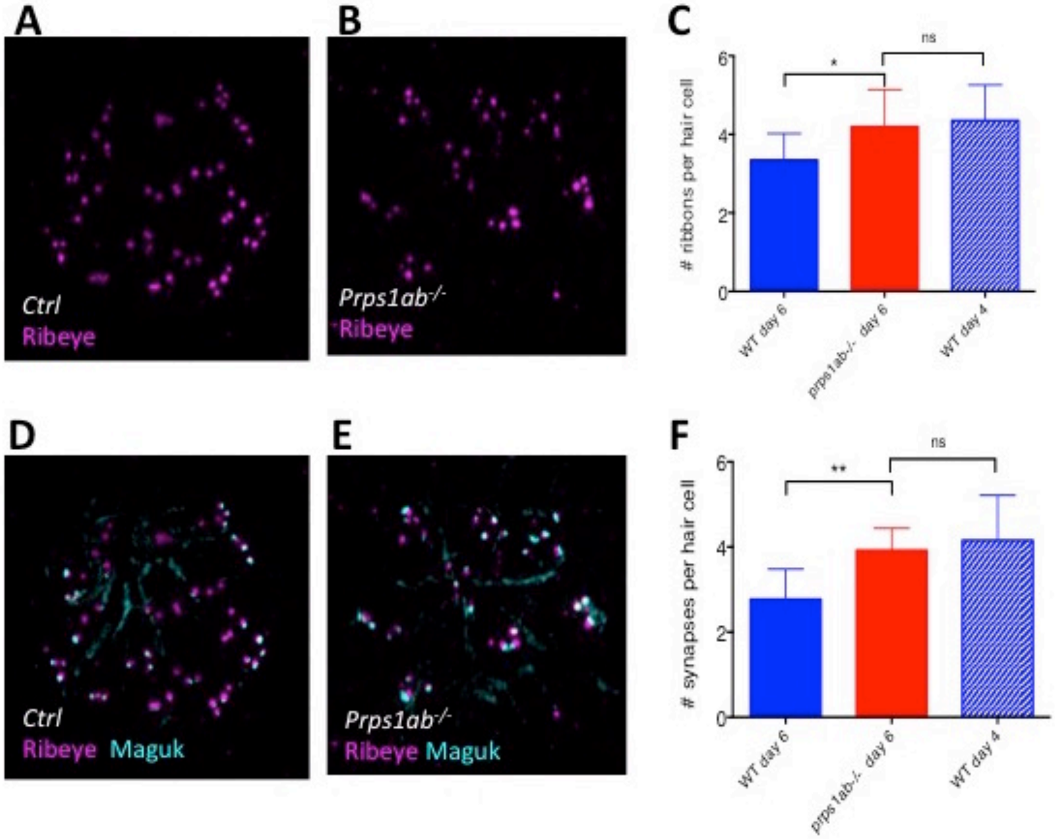

Suppl. Fig. 5. Analysis of presynaptic ribbons and synapse of neuromast hair cells in the double mutant.

(A-C) Increased presynaptic ribbons in the double mutants. (A, B) Morphology of Ribeye stained presynaptic ribbons of neuromast hair cells in the control (A) and double mutant (B) embryos at 6 dpf. (C) Quantification of the number of presynaptic ribbons in the control and mutant embryos at 6 dpf, as well as in wild-type embryos at 4 dpf. (D-F) Increased synapses in the double mutants. (D,E) Morphology of Maguk stained synapses and Ribeye stained presynaptic ribbons of neuromast hair cells in the control (D) and double mutant (E) at 6 dpf. (F) Quantification of the number of synapses in the control and mutant embryos at 6 dpf, as well as in wild-type embryos at 4 dpf. The numbers of neuromasts analyzed for each staining were 12, 13, and 10 for control, double mutant, and wild type embryos at 4 dpf, respectively.

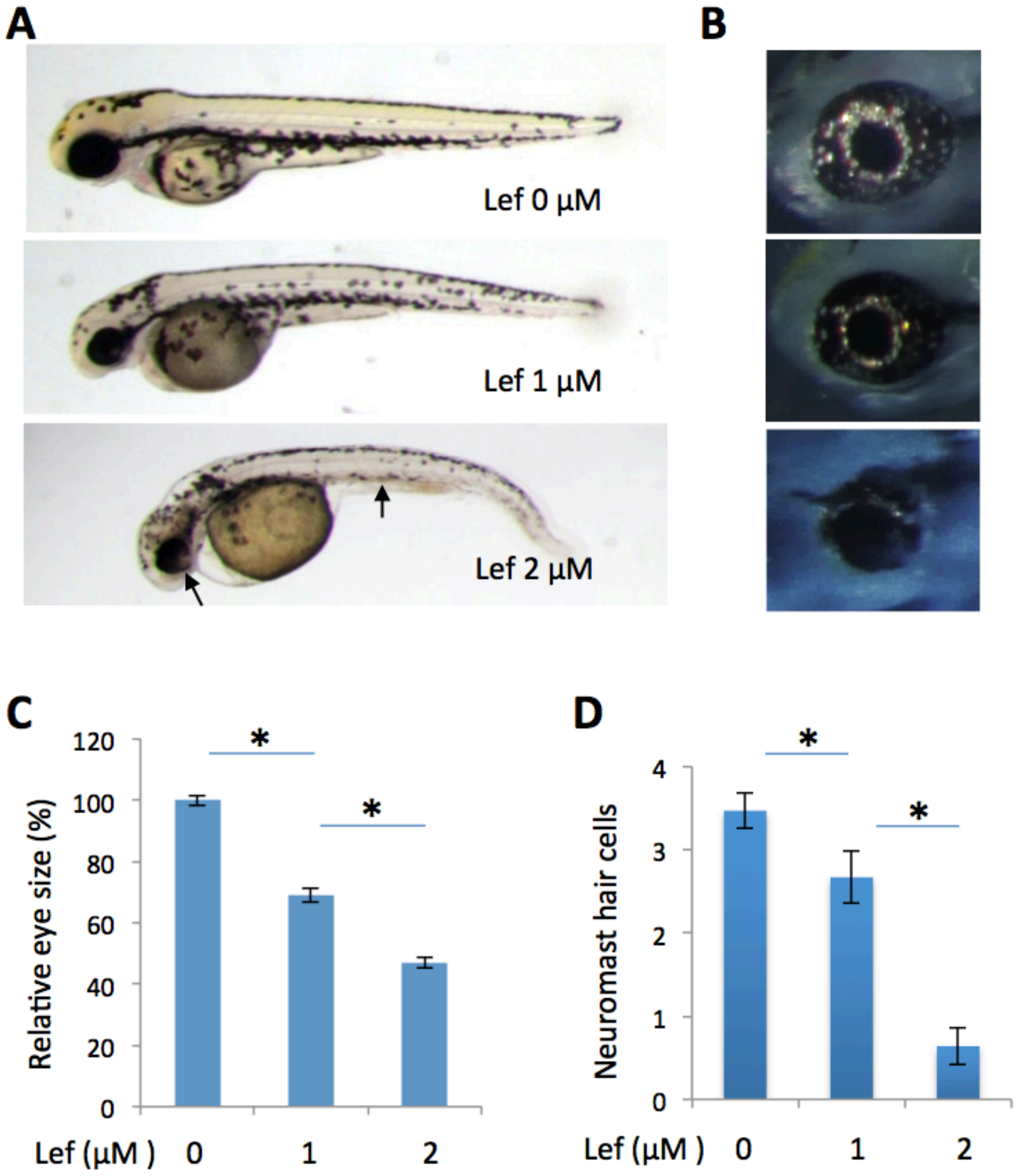

Suppl. Fig. 6. Inhibition on pyrimidine synthesis by leflunomide partially phenocopies the *prps1a:prps1b* mutant phenotypes.

(A) Leflunomide treatment of wild-type embryos leads to a dose-dependent reduction in melanocytes and eye size. Leflunomide concentrations are as labeled. Arrows point to small eye and melanocyte reduction in the embryo treated with 2 uM of leflunomide. (B) Dose-dependent reduction of retinal iridophores in leflunomide treated embryos. (C) Quantification of eye size

reduction. Eye size is measured by Image J. A significant reduction in eye size is detected between leflunomide at 0 uM and1 uM (n=10, p<0.001), and between leflunomide at 1 uM and 2 uM (n=10, p<0.001). (D) Dose-dependent reduction of neuromast hair cells in leflunomide treated embryos. A significant reduction in neuromast hair cells is detected between leflunomide at 0 uM and 1 uM (n=9, p=0.03), and between leflunomide at 1 uM and 2 uM (n=9, p<0.001). Graphs show the mean and s. e. m.. The data shown were replicated in another independent experiment.

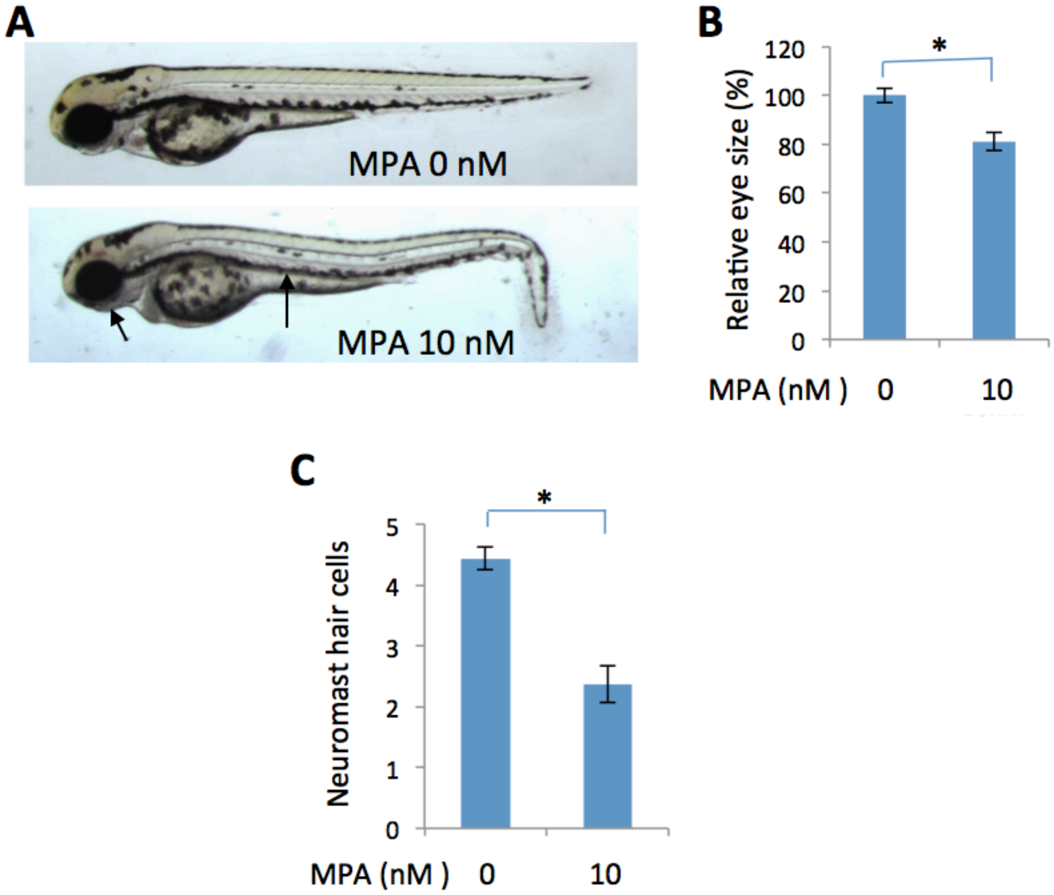

Suppl. Fig. 7. Inhibition on purine synthesis by mycophenolate mofetil partially phenocopies the *prps1a:prps1b* mutant phenotypes.

(A) Mycophenolate mofetil (MPA) treatment of wild-type embryos causes a reduction in melanocytes and eye size. Arrows point to small eye and melanocyte reduction in MPA-treated embryo. (B) Quantification of eye size reduction. Eye size was measured by Image J. There is a significant reduction of eye size in MPA-treated embryos (t-test, $p<0.001$). (C) Reduced neuromast hair cells in the treated embryos (t-test, $p<0.001$). Eye area and neuromast hair cell analysis were performed with 10 embryos for each group. Graphs show the mean and s. e. m.. The data shown were replicated in an additional independent experiment.

Suppl. Fig. 8

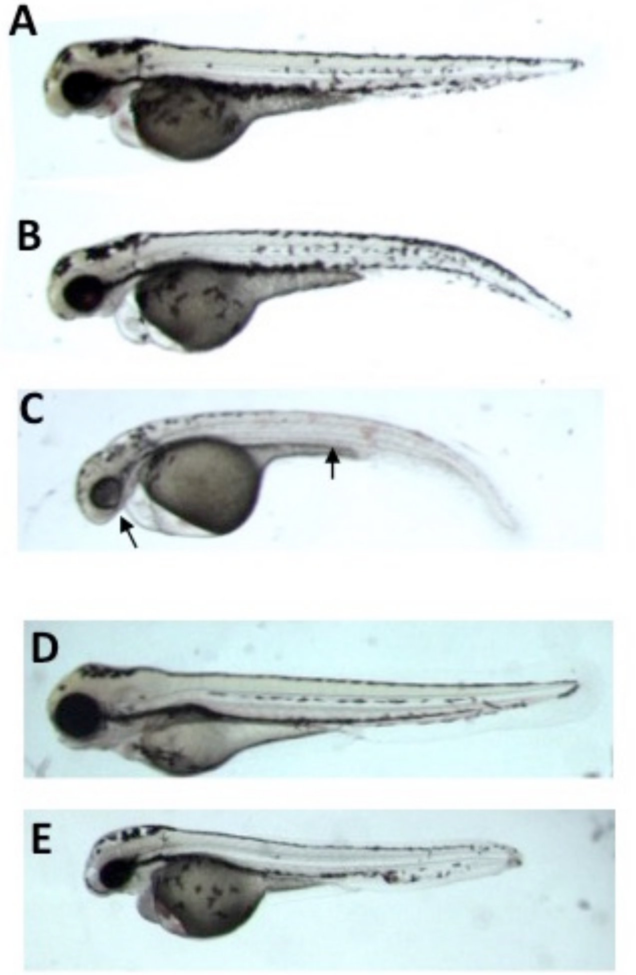

Suppl. Fig. 8. *prps1a:prps1b* double mutants are sensitive to inhibitions of nucleotide synthesis and ATP production.

(A-C) Morphology of embryos treated with 1 μM of leflunomide. A total of 76 embryos generated from a pairwise incross of adult fish carrying *prps1a* heterozygotic and *prps1b* homozygotic mutations were used for leflunomide treatment from 6 hpf until 3 dpf. Phenotype classification was performed at 3 dpf, with 19 embryos displaying a morphology as shown in panel A, 38 embryos as in panel B, and another 19 embryos as in panel C. Genotyping for *prps1a* mutation was conducted for 19 embryos from each class. The 19 embryos as in panel A included 8 wild-type and 11 heterozygotes. The 19 embryos as in panel B included 4 wild-type, 8 heterozygotes and 7 homozygotes. The 19 embryos as in panel C included 1 wild-type, 7 heterozygotes and 11

homozygotes. (D-E) Morphology of embryos treated with 0.5 ng/ml of antimycin A. A total of 32 embryos obtained as above were used for antimycin A treatment from 24 hpf until 3 dpf. Phenotype classification was performed at 3 dpf, with 24 embryos displaying a morphology as shown in panel D, and 8 embryos as in panel E. Genotyping for *prps1a* mutation revealed 11/11 embryos as in panel D were wild-type or heterozygotes for *prps1a*, and 8/8 embryos as in panel E were homozygotes for *prps1a*.

Suppl. Table 1. Somatic mutation analysis in *prps1a* CRISPR mutants.

Fluorescent PCR analysis was used to detect the *prps1a* mutations in the uninjected, Cas9 mRNA injected control, as well as Cas9 mRNA and prps1a gRNA co-injected *prps1a* CRISPR embryos. Size indicates the length (bp) of PCR amplicons. Height indicates the peak height of fluorescent signal. No wild-type amplicon was detected in either of the 10 *prps1a* CRISPR embryos.
